# Population genomics of grey wolves and wolf-like canids in North America

**DOI:** 10.1371/journal.pgen.1007745

**Published:** 2018-11-12

**Authors:** Mikkel-Holger S. Sinding, Shyam Gopalakrishan, Filipe G. Vieira, Jose A. Samaniego Castruita, Katrine Raundrup, Mads Peter Heide Jørgensen, Morten Meldgaard, Bent Petersen, Thomas Sicheritz-Ponten, Johan Brus Mikkelsen, Ulf Marquard-Petersen, Rune Dietz, Christian Sonne, Love Dalén, Lutz Bachmann, Øystein Wiig, Anders J. Hansen, M. Thomas P. Gilbert

**Affiliations:** 1 Centre for GeoGenetics, Natural History Museum of Denmark, University of Copenhagen, Copenhagen, Denmark; 2 Natural History Museum, University of Oslo, Blindern, Oslo, Norway; 3 Greenland Institute of Natural Resources, Kivioq 2, Nuuk, Greenland; 4 The Qimmeq project, University of Greenland, Manutooq 1, Nuussuaq, Greenland; 5 DTU Bioinformatics, Department of Bio and Health Informatics, Technical University of Denmark, Lyngby, Denmark; 6 Centre of Excellence for Omics-Driven Computational Biodiscovery, AIMST University,Kedah, Malaysia; 7 Sirius Dog Sled Patrol, Daneborg, Slædepatruljen SIRIUS, Greenland; 8 Greenland Wolf Research Program, Anchorage, AK, United States of America; 9 Department of Bioscience, Arctic Research Centre (ARC), Faculty of Science and Technology, Aarhus University, Frederiksborgvej 399, Roskilde, Denmark; 10 Department of Bioinformatics and Genetics, Swedish Museum of Natural History, Stockholm, Sweden; 11 Trace and Environmental DNA Laboratory, Department of Environment and Agriculture, Curtin University, Perth, Australia; 12 Norwegian University of Science and Technology, University Museum, Trondheim, Norway; National Institute of Genetics, JAPAN

## Abstract

North America is currently home to a number of grey wolf (*Canis lupus*) and wolf-like canid populations, including the coyote (*Canis latrans*) and the taxonomically controversial red, Eastern timber and Great Lakes wolves. We explored their population structure and regional gene flow using a dataset of 40 full genome sequences that represent the extant diversity of North American wolves and wolf-like canid populations. This included 15 new genomes (13 North American grey wolves, 1 red wolf and 1 Eastern timber/Great Lakes wolf), ranging from 0.4 to 15x coverage. In addition to providing full genome support for the previously proposed coyote-wolf admixture origin for the taxonomically controversial red, Eastern timber and Great Lakes wolves, the discriminatory power offered by our dataset suggests all North American grey wolves, including the Mexican form, are monophyletic, and thus share a common ancestor to the exclusion of all other wolves. Furthermore, we identify three distinct populations in the high arctic, one being a previously unidentified “Polar wolf” population endemic to Ellesmere Island and Greenland. Genetic diversity analyses reveal particularly high inbreeding and low heterozygosity in these Polar wolves, consistent with long-term isolation from the other North American wolves.

## Author summary

Full genome sequencing is becoming an increasingly valuable tool for both the management of animal populations, as well as fundamental to improving our understanding of their evolutionary history. The grey wolf (*Canis lupus*) is a keystone species in North America whose population structure and admixture has yet to be fully investigated in this way. We compiled a dataset of 40 full genomes spanning their total geographic range on the continent. In addition to confirming general population structure among them and previous reports of admixed origins for several wolf-like canid species, we identify three particularly interesting groups: two in Arctic Canada and one novel “Polar wolf” population on Ellesmere Island and Greenland. The particularly low genetic diversity of the Polar wolves suggests a small and isolated population. Overall we provide new information of relevance for the future management of wolves in Arctic Canada and Greenland.

## Introduction

Grey wolves (*Canis lupus*) currently occupy a wide range of habitats across North America, including the tundra, taiga, desert, plain, and boreal forest. Analysing ~40–50,000 SNPs from genotype arrays, the hitherto most comprehensive studies have identified seven North American grey wolf populations and ecotypes, which are referred to as West Forest, Boreal Forest, Arctic, High Arctic, British Columbia, Atlantic Forest, and Mexican wolves [1,2]. While this represents a major step forward in terms of describing the population structure, much remains to be learned. For example, nuclear DNA-based studies remain to include the full range of North American continental populations, omitting, for example, the Greenland wolves, despite mitochondrial DNA evidence suggesting it might represent an isolated population [3]. Furthermore, previous nuclear-DNA (nuDNA) based studies analysed SNP markers that were initially identified in the domestic dog (*C*. *l*. *familiaris*) [1,2]. Although dogs and wolves are closely related, phylogenetic analyses based on their nuclear genomes show that dogs are a distinct monophyletic clade within wolves [4–6]. Therefore, dog-ascertained markers may not be able to reveal the full genetic structure of wolves, and underestimate their true genetic diversity [7].

Outstanding questions also pertain to the taxonomic status of the North American wolf-like canids. These include the Southeastern red wolf (*C*. *rufus or C*. *l*. *rufus*) (subsequently referred to as the red wolf), as well as the Northeastern groups that are frequently referred to as Eastern timber wolves, Eastern wolves, Algonquin wolves or Great Lakes wolves (*C*. *lycaon or C*. *l*. *lycaon*) (subsequently referred to as the ‘Eastern timber/Great Lakes wolf’). While recent studies of both SNP-chip and whole genome resequencing data have shown that the genetic makeup of modern *C*. *l*. *rufus* and *C*. *l*. *lycaon* can be explained through admixture of various grey wolf and coyote populations [1,8,9], others argue for the possibility of a cryptic third ancestral canid species [10–12], sparking debate within the field of a two versus three species origin of *C*. *l*. *rufus* and *C*. *l*. *lycaon* [1,8,9,11–16]. Given this debate the definition and integrity of *C*. *l*. *rufus* and *C*. *l*. *lycaon* remains interesting, and clearly requires more research before the scientific community can agree on a fulfilling explanation for their origin and evolution.

In light of the above, we undertook an analysis of the genomic structure in, and admixture among, the full range of extant North American grey wolves, coyotes and wolf-like canids, by mapping the hitherto largest dataset of nuclear genome sequences against a *de novo* assembled wolf reference genome sequence [7]. To specifically test if wolves in Greenland are a unique population, and if the here analysed large genome data set potentially could bring further insight into the evolution of North American wolf like canids.

## Results

### Alignment, quality control and calling of genotype likelihoods

We generated resequencing data from 15 new canid samples, representing 13 North American grey wolves, one red wolf and one Eastern timber/Great Lakes wolf. Between 56 and 400 million paired end reads were generated per sample. After quality control, including removal of adapters, discarding of low quality reads and removal of duplicates, these reads were aligned to the *de novo* wolf reference genome [7], which resulted, for most of the samples, in depth of coverage between 3.8–15.3x. The exception being the ‘Krummelangsø’ wolf from Greenland, with coverage of only 0.4x. We complemented this dataset with 25 previously published samples, all of which were re-mapped following our mapping pipeline and yielded genomes with coverages of 2.1–26.4x. Additional details for the samples can be found in supplementary S1 Table.

The error rates estimated for the different samples (S1 Table and S1 Fig) were estimated in *ANGSD* [17], using the ‘Daneborg’ Greenland wolf sample as the “error-free” model sample and the ‘Golden Jackal’ as the outgroup. For the newly sequenced samples the error rates ranged between 0.039–0.076%, except for the ‘Krummelangsø’ Greenland wolf whose error rate was 0.146%, consistent with lower sequencing coverage. We also noted elevated error rates in the data from several of the previously published samples (0.146%-0.636%), including three coyote samples (‘Illinois’, ‘Quebec’ and ‘Alabama’), the ‘Red wolf 2’ sample, and the wolves ‘Eurasia 3’ and ‘Yellowstone 1’. Because error rates can affect the results of some analyses, for example the terminal branch lengths estimated using Treemix, they must be considered when drawing conclusions from the results.

### Structure and admixture

**Fig 1:**
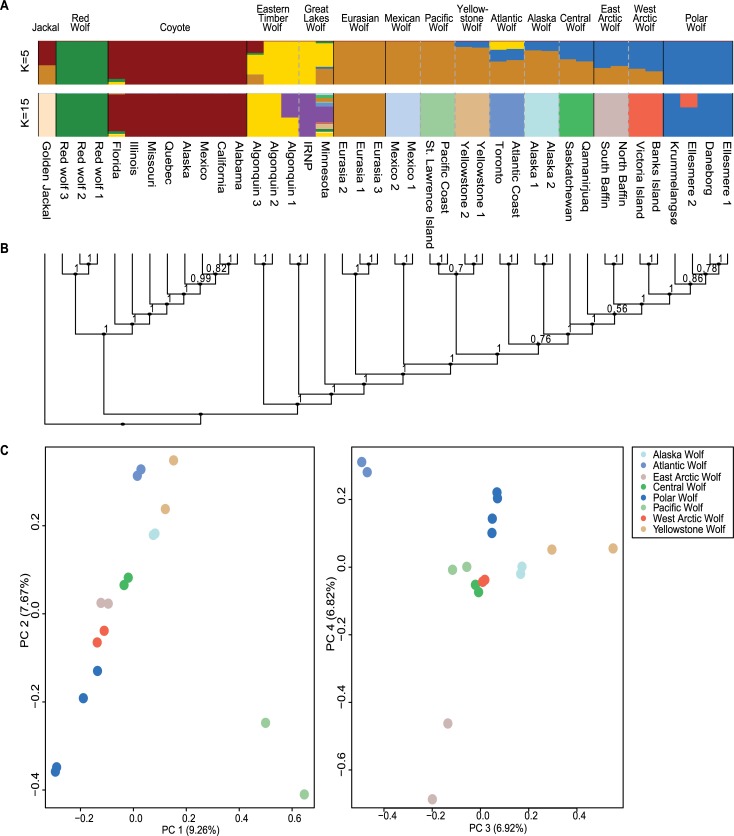
Population structure of North American wolves and wolf-like canids. **A**) Ancestry proportions estimated using *NGSadmix* for K = 5 and K = 15 clusters. **B**) Astral phylogeny of the 40 samples in the study, plotted to coincide with the admixture plot above, with the local posterior probabilities, computed in Astral, shown at the nodes. **C**) Principal component analysis of grey wolf individuals identified in the data, with colours matched to cluster assignment in the admixture plot.

When inferring ancestry clusters using admixture, with two ancestry clusters (K = 2), all samples split into two separate clusters representing the grey wolf-like and coyote-like (S2 Fig). When the number of clusters is increased to three (K = 3), the grey wolves subdivide into one cluster represented by Polar wolves, and a second cluster represented by Eurasian, Mexican and Pacific wolves. All other wolf lineages derive from these two clusters. At K = 4, the red wolves split from coyotes, and at K = 5, Eastern timber/Great Lakes wolves form their own cluster (Fig 1A), while the wolves remain as two additional clusters, one containing the Eurasian, Yellowstone, Mexican and Pacific wolves, and the other represented by the East Arctic, West Arctic and Polar wolves. The remaining wolves are mostly represented as a combination of ancestries from these two wolf clusters. However, some wolves showed low levels of shared ancestry with the other three non-grey-wolf clusters. As we increased the number of clusters to K = 15 (Fig 1A), a pattern emerged that is consistent with both the results of the phylogenetic reconstruction and the PCA, making us choose K = 15 at the upper justifiable number of ancestry clusters. Grey wolves split into 9 clusters, each identifying a population of North American wolves, specifically: (1) Mexican, (2) Pacific, (3) Yellowstone, (4) Central, (5) Alaskan, and (6) Atlantic wolves, as well as three groups from the high Arctic, namely (7) West Arctic (representing the Banks and Victoria Islands), (8) East Arctic (representing the Baffin Islands), and (9) Polar (representing Ellesmere Island and Greenland). The (10) red wolves, (11) coyotes and (12) Eurasian wolves each grouped into separate clusters, while individuals from (13) the Algonquin Provincial Park formed a cluster that is henceforth referred to as to as Eastern timber wolves. The samples from (14) Isle Royale National Park and Minnesota formed a cluster referred to as Great Lakes wolves, that is closely related to the Eastern timber wolves. The final cluster (15) contained the ‘Golden Jackal’ outgroup.

Phylogenetic reconstruction based on 40 nuclear genomes (Fig 1B and S3 Fig) revealed three major clades: one containing the ‘Golden Jackal’ outgroup, a second containing the red wolves and coyotes, and a third containing all grey wolves together with Eastern timber/Great Lakes wolves. These observations of affinity between red wolves and coyotes, and Eastern timber/Great Lakes wolves alongside grey wolves, were also supported by the admixture, Treemix and D-statistics. Within the wolf clade, we observed Old and New World wolves to be reciprocally monophyletic, and within the New World grey wolves, we found the Mexican wolves to be the most divergent from all others. Although we note that (i) the overall phylogenetic relationships between the golden jackal, coyotes and grey wolves, and (ii) the divergence of Mexican from other New World wolves, were recovered in a previous nuDNA-based analysis [4], the inclusion of additional North American samples improved the resolution of these relationships. Specifically, it was noted that after the aforementioned basal divergence of Mexican, Yellowstone and Pacific wolves, the remaining North American populations formed a monophyletic clade. Surprisingly, the 2 individuals identified as Central wolves did not form a clade; the Saskatchewan individual was basal to the one from Qamanirjuaq Lake (Nunavut), which in turn was sister group to the remaining Arctic and Polar wolves. However, the position of the Qamanirjuaq individual is only poorly supported. Given that admixture and PCA analyses indicate that its genetic background is largely similar to the Saskatchewan individual, we believe its phylogenetic placement is likely the result of gene flow from other Northern wolf populations. We caution, however, that any conclusions drawn from the phylogenetic tree must be tempered by the large amounts of allele sharing observed in the population genomic analyses (D-statistics, Admixture and Treemix). Further, the amount of incomplete lineage sorting between the different wolf populations that relates to their recent divergence from each other, suggests that several equally likely alternative placements exist for many of these nodes (S3 Fig).

Principal component analyses were used to project the SNP variation of the wolves in two dimensions (Fig 1C, S4 Fig and S5 Fig). The wolf diversity expressed in PC1 vs. PC2 (variance explained 9.26–7.76%), and PC3 vs PC4 (variance explained 6.92–6.82%) (Fig 1C) clearly showed a signal that correlates with the geographical distribution of samples running North-South and East-West. Polar, Pacific and Atlantic wolves exhibited highest variation in PC1 and PC2. Furthermore, Polar and East Arctic wolves were also clearly distinct in PC3 and PC4. The grouping of individuals was congruent with the clusters identified by *NGSadmix* [18], and the tree topology delineated in the phylogenetic reconstruction.

Evidence of gene flow among the North American canids was obtained from the D-statistic analyses on the genomes (S6 Fig and S7 Fig). A test of coyote ancestry among the different North American canids (S6 Fig) revealed that all North American wolf-like canid populations had a significant, but varying, degree of coyote ancestry, consistent with previously published findings [8,9]. Specifically, the highest levels of coyote ancestry were observed in the red wolves, and somewhat lower levels were found in the Eastern timber/Great Lakes wolves. Lowest, although still identifiable, values were observed in the Mexican and the Atlantic wolves. Our expanded dataset also enabled testing for gene flow between North American and Eurasian wolves. The results indicated gene flow between the East Siberian (Chukchi) wolf ‘Eurasia 2’ and the Alaskan wolves, consistent with their geographic proximity (S7 Fig).

Treemix analyses (S8 Fig, S9 Fig and S10 Fig) yielded results that were consistent with the phylogenetic reconstruction (Fig 1B and S3 Fig), with migration events indicating allele sharing between the wolf-like canids, and likely shared coyote ancestry in the Yellowstone, Mexican and Pacific wolves.

**Fig 2:**
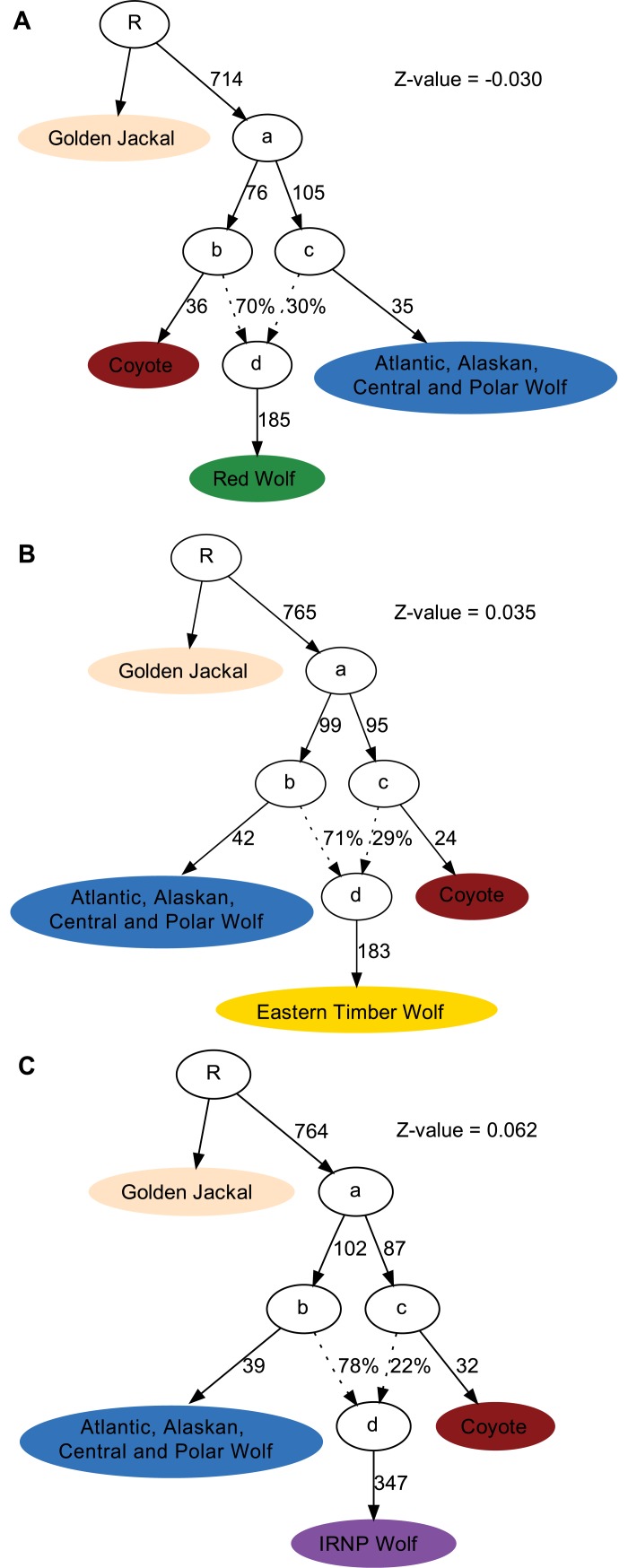
Admixture graph modelling the origin of American wolf like canids. Models fitting the ancestral makeup of **A**) red wolves, **B**) Eastern timber wolves and **C**) Great lakes wolves. The specific samples used in each cluster are given in S1 Table. Internal nodes denoted by letters from a to d are hypothesised meta-populations. Tip nodes indicate the sampled genomes used to fit the graph. Dotted connecting lines represent admixture events, with the percentages indicating the admixture proportions. Solid connecting lines represent the divergence between populations with the numbers indicating their corresponding branch lengths.

**Fig 3:**
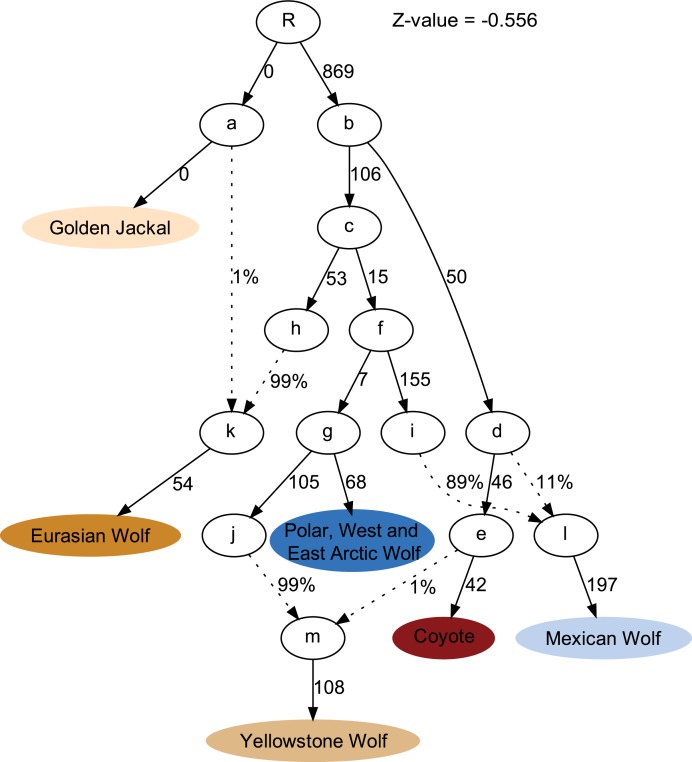
Admixture graph modelling the origin of Mexican wolves. Lowest fitting admixture graph for the formation of Mexican wolves, the specific samples used in each cluster are given in S1 Table. Internal nodes denoted by letters from a to m are hypothesised meta-populations. Tip nodes indicate the sampled genomes used to fit the graph. Dotted connecting lines represent admixture events, with the percentages indicating the admixture proportions. Solid connecting lines represent the divergence between populations with the numbers indicating their corresponding branch lengths.

Using admixture graphs (Fig 2), we modelled the genomic makeup of red, Eastern timber and Great Lakes wolves, as composed of genomic variation found in North American grey wolves and coyotes. When using admixture graphs (Fig 3) and (S11 Fig) to investigate the relationships between Eurasian, Mexican and other North American wolves, the best fitting graph (Z = -0,556) assigns Eurasian wolves as sister to all North American wolves, with the Mexican wolf sister to other American wolves, containing considerable coyote introgression. The most parsimonious explanation for this outcome is that all extant North American grey wolves descend from the same ancestral wolf diversity, although whether this ancestral “population” had colonised the North American continent prior to, or post (possibly on multiple occasions) the divergence between Mexican and other North American wolves remains a open question.

**Fig 4:**
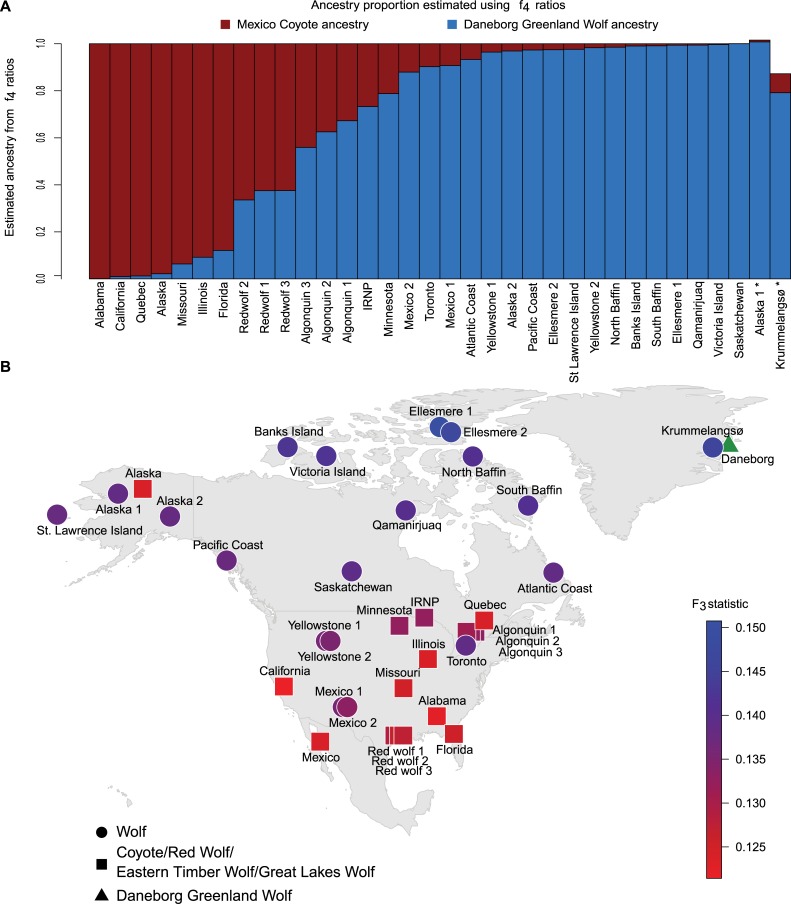
Genetic affinity and admixture proportions. **A**) Wolf vs. coyote ancestry proportions estimated from f4 ratios, using the ‘Daneborg’ Polar wolf, and the ‘Mexico’ coyote as representatives of the two groups respectively. * indicates samples with erroneous estimates, either due to closeness to ‘Eurasia 1’ (‘Alaska 1’), or paucity of data (‘Krummelangsø’). The f4 ratios can be used to quantify the amount of admixture from different source groups, based on the sharing of alleles, as computed by the f4 statistics [59]. **B**) Genetic affinity of each sample to the ‘Daneborg’ Polar wolf, computed as the f3 statistic using the ‘Golden Jackal’ as the outgroup. The symbols are plotted on a blue-red scale, where blue indicates higher affinity and red indicates lower affinity. Circles represent grey wolves and squares indicate wolf-like canids.

We used f4 ratios to investigate proportion of coyote and grey wolf ancestries in the North American wolf-like canids, setting aside the Polar wolf (‘Daneborg’) and coyote (‘Mexico’) as references (Fig 4A). These samples were chosen based on their respective distance to the coyote or wolf cluster in the PCA (S4 Fig), which suggests they may represent the “purest” examples of coyote and North American wolf in our dataset. The f4 ratio estimates showed that the coyotes from Alabama, California, Quebec and Alaska harbour negligible wolf ancestry, while those from Missouri, Illinois and Florida contained between 5–10% wolf ancestry. Much higher levels of wolf versus coyote admixture were observed in red wolves (40%:60%), the Eastern timber wolves (60%:40%), and the Great Lakes wolves (75%:25%). Within wolves, coyote ancestry was highest in the Mexican wolves and the Atlantic Coast wolves (10%), followed by the Pacific Coast and Yellowstone wolves (~5%). The wolves from the Canadian archipelago showed less than 3% coyote ancestry. The higher than 100% combined admixture proportions estimated for the wolf ‘Alaska 1’, likely result from the tree configuration, with the ‘Eurasia 1’ wolf being a fixed member of the quartets used to compute the admixture proportions and indicate Eurasian wolf gene flow into ‘Alaska 1’, something also supported by D-statistics (S7 Fig). The admixture proportion estimates do not need to add up to 100% because they are estimated separately for the ‘Daneborg’ wolf and the ‘Mexico’ coyote component. Nevertheless, nearly all estimates summed up to 100%, indicating that most samples can be modelled as a mixture between just two components, the wolf and the coyote. f3 statistics were also computed to assess the affinity of the various North American wolf-like canids to the ‘Daneborg’ Polar wolf. As expected from their geographic proximities, wolves from the Canadian Arctic archipelago displayed the highest affinity (Fig 4B), while the amount decreased in populations from further West and South. Furthermore, populations such as the Eastern timber/Great Lakes and red wolves that had substantial amounts of coyote ancestry, showed the lowest affinity with Polar wolves. An inverse pattern was observed when affinities were assessed with the ‘Mexico’ coyote, yielding lowest coyote affinity with the most Northern and Eastern populations (S12 Fig).

### Heterozygosity, inbreeding and runs of homozygosity (ROH)

**Fig 5:**
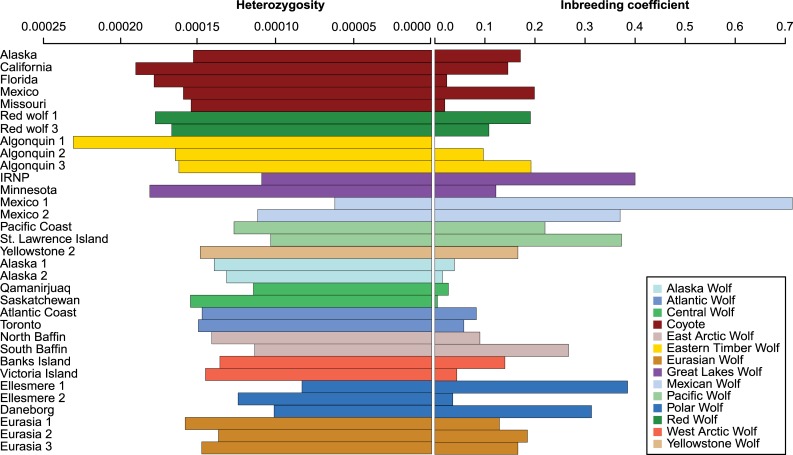
Bar charts of heterozygosity estimates and inbreeding coefficients. To the left, estimates of heterozygosity estimated using ANGSD, obtained by bootstrapping the set of variant sites. The standard errors are of the order of 10^–6^ (not shown on plot). The colours represent the different population of North American wolf like canids.

Our pan-population dataset also enabled us to undertake the first whole-genome based, continental-scale investigation of heterozygosity and inbreeding levels in these canids (S1 Table). The 6 samples with highest estimated error rates (marked with *, S1 Table) also have the highest estimates of heterozygosity and low inbreeding coefficients. Given the error rate, heterozygosity and inbreeding coefficients must be interpreted with care in these individuals. The estimates for the remaining grey wolves, coyotes and wolf-like canids (Fig 5) allow for more robust interpretation. The heterozygosity estimates indicated that higher diversity exists among the coyotes, red wolves and Eastern timber/Great Lakes wolves, than in any of the North American grey wolf populations (Fig 5). Further, within the “true” wolves, the Polar and Mexican wolves showed the lowest heterozygosity, while the Eurasian wolves had the highest. In order to estimate the inbreeding coefficients for these samples, we split the samples into 2 groups, as indicated by the phylogeny, i.e. the red wolves and the coyotes in one group, and the Eastern timber/Great Lakes wolves and the grey wolves in another. To avoid overestimating the inbreeding coefficients (caused by the Wahlund effect), we estimated the allele frequencies in each of these clusters separately, and used these allele frequencies to estimate inbreeding coefficients. Overall, values of inbreeding were relatively low, and the highest values were obtained for the Mexican, Pacific, and one Great Lakes (Isle Royale National Park) wolf (0.2<F<0.7) (Fig 5). The ‘Ellesmere 2’ Polar wolf showed rather low (0.1<F) levels of inbreeding, which we ascribe to likely admixture (Fig 1A). The ‘Daneborg’ and ‘Ellesmere 1’ Polar wolves showed higher (F<0.5) levels of inbreeding, which is probably a more accurate representation of the inbreeding levels in the “Polar wolf” population.

To further examine the levels of inbreeding, we estimated the fraction of the genome in long runs of homozygosity (ROH) on a subset of seven selected wolves with high coverage, including the ‘Daneborg’ Polar wolf (S13 Fig and S1 Table). The Mexican wolf—Mexico 1—showed the highest proportion of the genome contained in ROH longer than 1 Mb, followed by IRNP and Daneborg. The Polar wolf contained more than ~15% of its genome in long ROH, but none of these segments were longer than 4 Mb, in contrast to IRNP and Mexico 1, which contained ROH segments longer than 6 Mb. When comparing Daneborg to East and West arctic wolves, represented by Banks Island and North Baffin respectively, the Polar wolf showed significantly longer and more abundant ROH, implying higher levels of inbreeding in the Polar wolf compared to its Arctic conspecifics.

## Discussion

Our analyses are based on a full genome dataset spanning the full range of extant North-American wolves, coyote and wolf-like canid populations. Therefore, our results both complement, and expand beyond the conclusions of previous studies [1,2,4,8,9,12,19], principally through refining prior observations, reconstructing new population structure, and providing detailed insights into admixture levels and the diversity within and between the populations. Perhaps most importantly we report the first Polar wolf genomes, which enabled us to obtain insights into that population’s discrete genetic status.

While potentially restricted by the specific sample sizes, sample level heterozygosity and inbreeding estimates across the data set, in combination with ROH estimates for seven samples (S1 Table), offer interesting insights into the population history of the North American canids. In general, coyotes, red wolves and Eastern timber/Great Lakes wolves show high amounts of heterozygosity and low levels of inbreeding, with the notable exception of the Isle Royale National Park wolf (IRNP). This individual is from a population famous for extremely high levels of inbreeding leading to deformities and low fitness [20–23]. Interestingly several wolves from other populations also show similar values of heterozygosity and inbreeding, viz., a Pacific wolf (St. Lawrence Island) and two Polar wolves (Ellesmere 1 and Daneborg). Both of these populations are isolated on islands and thus likely have low population sizes. This is in striking contrast to the continental populations of Alaskan, Central and Atlantic wolves, which display low inbreeding coefficients likely due to being larger populations and connections to neighbouring populations. One Polar wolf (Ellesmere 2) has a notably low inbreeding coefficient when compared to the other Polar wolves, this corresponds well with the observation in NGSadmix (Fig 1), that the individual is admixed with the West Arctic wolves. Similarly, the Mexican wolves show signatures of low population sizes, with low heterozygosity and high inbreeding coefficients, with Mexico 1 estimated to have the highest inbreeding coefficient in the entire dataset. This corresponds with expectations based on a founding population of 4–5 individuals [24,25]. Intriguingly, the ROH analysis (S13 Fig), summarizing the genomic signatures of inbreeding, places the Polar wolf as intermediate between the highly inbred Mexican and IRNP wolves, and the remaining continental wolves with low levels of inbreeding. Given the unique breeding history of both the Mexican and IRNP wolves, this leaves the Polar wolf as the individual from a “natural” population with longest ROH, indicating more recent inbreeding than in the other samples with high inbreeding coefficients. Interestingly, although the red wolves also went through a severe bottleneck which can be traced back to 14 founders [26,27], they show high heterozygosities and low inbreeding coefficients. The admixed ancestry of the red wolves (Fig 1, Fig 2 and Fig 4) might explain the higher diversity of this population. While severe inbreeding can lead to inbreeding depression, genomic meltdown and eventually extinction [28,29], the inbreeding values presented here should be interpreted with care, due to both the low sample sizes and low genomic coverages of some of the samples. Therefore we are limited in estimating the effects of inbreeding in these populations. Rather, the observed levels of heterozygosity and inbreeding coefficients offer a qualitative insight into the demographic processes of these populations, in terms of isolation, connectivity and bottlenecks, and thereby capture the legacy of specific population histories in the individual genomes.

At the continental scale, and consistent with the results of previous studies, we found weak yet visible support for an East-West structure in coyotes [1,12,30]. One of the clades principally contained animals sampled in the west of the continent (including animals from Mexico, California and Alaska, but also notably a coyote from as far east as Alabama). However, there was no monophyletic ‘Eastern clade’, which is likely due to varying levels of admixture with red, Eastern timber/Great Lakes and grey wolves. Nonetheless, coyotes from Florida, Illinois, Missouri and Quebec do cluster together in our analyses (S2 Fig and S4 Fig).

Whether the evolutionary history of red wolves, Easter timber wolves and Great Lakes wolves is genetically elucidated, is a matter of contrasting opinions within the scientific community [1,8,9,11–16]. The essence of the debate is whether the formation of the wolf-like canids arose from admixture between two or three canid species, the former being grey wolf and coyote, while the latter including also a now extinct (and unknown) third canid species. The debate is complex, yet broadly concerns two issues. Firstly, whether or not the samples investigated to date are relevant representatives of each wolf-like species. Secondly, that while grey wolf and coyote derived ancestry in all wolf-like canids is well proven, whether ancestry of a potential third canid “species” can be soundly rejected.

With regards to the first issue, it should be noted that our analyses rely partly on previously published Eastern timber/Great Lakes wolf genomes [9,31]. Hohenlohe and colleagues have expressed concern about whether these published Eastern timber wolves (Algonquin wolves) truly represent the wolf-like canid in question [15]. In this regard, our provision here of a third Eastern timber wolf genome (Algonquin 3) collected in Algonquin Provincial Park 2010 (S1 Table) provides relevant information. This new sample, clusters together with one of the previously published Eastern timber wolves (Algonquin 2), while the second previously published sample (Algonquin 1) shows evidence of admixture with the Great Lakes wolves. Thus while full resolution of the question as to whether samples Algonquin 2 and 3 are authentic Eastern timber wolves will require a larger and nuclear genome dataset of canids from the Algonquin Provincial Park area, we believe it is fair to assume that samples Algonquin 2 and 3 are the best currently available representatives of this wolf-like canid lineage. Thanks to the analytical power conferred by our whole genome data, we were also able to reconstruct how the coyote and grey wolf lineages have contributed to the genomic makeup of the wolf-like canid specimens and populations analysed here. In this regard, and of direct relevance to the question of two vs three species origin of the wolf-like canids, we find our results are consistent with previous conclusions [8,9]. Specifically, that the red wolf and Eastern timber/Great Lakes wolves can be explained through admixture of modern coyote and modern North American grey wolf lineages. Interestingly however, both groups showed individual, though largely consistent levels of wolf versus coyote genetic makeup, which suggests they may have formed through relatively old hybridization events. Furthermore, in the admixture analyses, the red wolves were one of the first groups to be assigned a separate cluster, indicating a large amount of drift in this lineage, which may reflect historical population bottlenecks prior to captive breeding of the modern population [27,32]. The Eastern timber/Great Lakes wolves also differed from other populations, indicating the presence of population specific variation in these samples. Interestingly, there were two separate populations of “Eastern timber” and”Great Lakes” wolves. This observation is admittedly based on small sample sizes. More data will be required to address whether this isolation has persisted over longer time spans, or if it reflects different patterns of genetic drift in the isolated subpopulations after recent bottlenecks. However, the observation that the ‘Algonquin 1’ individual is admixed with both the Eastern Timber and Great Lakes wolves clusters, indicates recent contact and admixture between these populations. Overall therefore our analyses support a coyote-grey wolf admixture origin to the wolf-like canids, followed by subsequent structural development at the specific population level. This raises the natural question as to whether our findings can be used to solidly reject a third “species” ancestry, that is including an as yet unidentified distinct extra canid? The results of our admixture graph analysis (Fig 3) are helpful in supplementing the results of previous studies this regard. Specifically, given that the ancestry of red, Eastern timber and Great Lakes wolves can be fully explained by combining ancestry of modern coyotes and grey wolves, ancestry of a third distinct lineage is only likely if that lineage had also introgressed into the reference coyotes and grey wolf samples. If this was so, then this third lineage would have also played a role in the formation of the modern coyote and/or North American grey wolves. Interestingly there may be some evidence in support of a third potential canid lineage in North America, given the distinct Y-chromosomal and mitochondrial diversity found in some wolf-like canids—especially in the Eastern timber/Great Lakes wolves complex [10,30,33–37]. Ultimately however, full resolution to the evolutionary history of grey wolves, coyotes and wolf-like canids in North America, may require data from a large number of ancient genomes with broad temporal and geographic context.

Based on our analyses, it is clear that Mexican wolves are divergent from all other North American wolf populations, and given they form a sister group to all other populations regardless of how they are analysed, they have likely been isolated from other grey wolf populations represented in this study. This divergence is well described, and hypotheses to explain this could be that their presence in the Americas arises from a different colonization history to that of the remaining North American grey wolves [1,4,24,38]. An alternative explanation could be that Mexican wolves diverged early on in a single colonisation event, and have since been isolated from the other populations. In addition, Mexican wolves carry substantial coyote admixture. The admixture from coyotes could also play a role in the basal phylogenetic placement of the Mexican wolves. Similar levels of coyote admixture are present in Atlantic wolves, but do not have the same phylogenetic impact. The wolf diversity in Atlantic wolves seems closely related to diversity in neighbouring wolf populations, giving the lineage affiliation with other Northern American wolves. However the Mexican wolves have no surviving neighbouring wolf populations, a factor further contributing to their distinctness compared to the available references. While clearly distinct, we find that Mexican wolves have the same cladistic ancestry as other American grey wolves, and note that ancient samples will be highly relevant in addressing whether the last common ancestors of North American wolves were within or outside the continent.

It is also clear that while Eurasian wolves are a sister clade to all North American grey wolves, the two groups are not completely reproductively isolated. For example analyses using D-statistics revealed some inter-continental admixture between the populations represented by a Eurasian Chukchi and Alaskan wolves (S6 Fig). The inclusion of genomic data from wolves from the high Arctic Canadian archipelago and Greenland also provided key insights that may become relevant for the future management of these populations. Firstly, there was evidence for three genetically distinct populations referred to as the Western Arctic, Eastern Arctic and Polar wolves. Although our phylogenetic analyses indicate that East Arctic wolves constitute a sister group to a monophyletic cluster containing West Arctic and Polar wolves, PCA and Admixture analyses indicated that Polar wolves constitute a distinct population (Fig 1, S2 Fig and S4 Fig). The distinctness of the Polar wolf cluster is probably due to a greater genetic overlap between the East Arctic, West Arctic and Central wolves populations, pulling the Arctic populations closer to the mainland population. It is important to note, however, that the Polar wolf population of this study is represented only by contemporary samples from Greenland. It is currently believed that wolves were most likely exterminated in East Greenland in the 1930s, and have only returned slowly since—wolf sightings did not become frequent until the 1970s [39,40]. Therefore it is possible that the Greenlandic wolves included in this study are recent immigrants from the neighbouring Ellesmere Island. Therefore, they may not accurately reflect the gene pool of historic East Greenland wolves. However, if East Greenland was originally home to a fourth high Arctic population, then there was at least no evidence of it as an additional ancestry component potentially surviving as part of the modern Polar wolves. To address this issue will require the analysis of genomes recovered from pre-1930s Greenland wolves.

Our whole genome-based analyses reconstructed the overall genetic structure of the North American grey wolf populations. In extension to the results of previous studies [1,2,41–44], the Polar wolves from Ellesmere Island were found to be genetically different from the wolves from Victoria and Banks Island (West Arctic wolves). Schweizer et al. [2] reported these two populations are genetically similar, but this discrepancy may either reflect that each study sampled different populations from Ellesmere Island (we were unable to confirm this through the identity of metadata tied to the samples), or an artefact introduced through the genetic markers used in the two studies. We note that while Schweizer et al. [2] analysed ~40,000 dog specific SNPs typed using the Affymetrix v2 Canine SNP array, while the genome dataset used in this study, was mapped against a wolf reference genome, contained ~4M SNPs. In light of the results of prior analyses specifically undertaken on the draft wolf genome to explore this matter [7], we believe that these ~4M North American grey wolf, coyote and wolf-like canid specific SNPs are less biased, thus more informative, than the dog specific SNPs used in previous studies. Therefore, our findings of a previously undetected structure in high arctic wolves may imply that markers identified in dogs are inadequate for in-depth investigations of population structure in wolves.

### Conclusion

Whole genome sequencing of North American grey wolves and wolf-like canids showed complex mixing of the wolf and coyote lineages. We find the ancestral genomic makeup in the controversial red, Eastern timber and Great Lakes wolves, can be explained as admixture between modern grey wolves and coyotes. However, there were also population specific divergences in these lineages, which distinguish them from modern wolves and coyotes. All in all—to explain modern genomic structure, if a third cryptic canid species have been involved in the formation of the wolf-like canids, this lineage must also be admixed into modern coyotes or grey wolves. Finally, three distinct grey wolf populations were identified among high arctic wolves, including a novel and highly distinct Polar wolf population endemic to Ellesmere Island and Greenland. Overall, our study provides results for future research in canid evolution and relevant knowledge about North American grey wolves and wolf-like canids.

## Material and methods

### Data

Our dataset consists of 25 previously published canid genomes, 21 of which are derived from North American grey wolves, coyotes, wolf-like canids and a golden jackal (*Canis aureus*) [4–6,9,31], as well as new data from 15 additional New World canid specimens sequenced to a coverage of between 0.4 and 15x These additional samples consist of one red wolf, one Eastern timber/Great Lakes wolf and 13 grey wolves. Four of the grey wolves are from the High Arctic. Details on samples can be found in supplementary S1 Table and Fig 4B. Samples originating from Canada or the USA were obtained under Article VII, paragraph 6 CITES convention for import as scientific exchange between CITES institution Natural History Museum of Denmark (DK-003), U.S. Fish and Wildlife Service (US 096 (A/P)), University of New Mexico Museum of Southwestern Biology (US 119 (A/P)), University of Alaska Museum of the North (US 130 (A/P)) and University of Alberta Museums & Collection Services (CA-010). DNA was extracted using the DNeasy Blood & Tissue Kit (Qiagen) following the manufacturer’s protocol. DNA was converted into double stranded blunt-end libraries with Illumina-specific adapters [45] using the NEBNext DNA Sample Prep Master Mix Set 2 (E6070S - New England Biolabs Inc., Beverly, MA, USA) following the manufacturer’s protocol. Libraries were sequenced on Illumina HiSeq 2500 platforms using 100 base pair paired-end read chemistry.

### Quality control and alignment

The short-read data from each sample (including the previously published genomes) were mapped against a recently published wolf reference genome [7]. The *PALEOMIX* (v1.2.5) [46] pipeline was used to process the reads and to remove adapters. Subsequently, the reads were mapped to the reference genome using the *bwa* (v0.7.10; *aln* algorithm) [47]. Picard (v1.128, https://broadinstitute.github.io/picard) was used to exclude reads that were PCR or optical duplicates, and to exclude reads that mapped to multiple locations in the genome. *GATK* (v3.3.0) [48,49] was used to perform an *indel* realignment step to adjust for increased error rates at the end of short reads in the presence of *indels*. In the absence of a curated dataset of *indels* in wolves, this step relied on a set of *indels* identified in the specific sample being processed.

### Calling of genotype likelihoods

The samples in this study have very disparate coverages across the genome. Instead of calling genotypes at variant sites, which have been shown to introduce biases [50], the uncertainty in genotypes was propagated to downstream analyses using genotype likelihoods. The genotype likelihoods at variant sites were computed in *ANGSD* (v0.919) [17] using the aligned reads obtained from *PALEOMIX*, under the model proposed in *samtools* (v1.2) [47]. Nucleotides with base qualities lower than 20 and reads with mapping quality lower than 20 were discarded. Sites with coverage at fewer than 38 out of the 40 samples were excluded. Finally, only sites with an estimated minor allele frequency greater than 0.05 were retained.

### Admixture analysis

Clusters of ancestry and the associated ancestry proportions were estimated using *NGSadmix* [18] taking into account the genotype likelihoods obtained from *ANGSD* [17]. Since low frequency markers are uninformative for admixture analyses, only markers with minor allele frequency greater than 0.1 were used for this analysis, which resulted in a total of approximately 4.47 million SNPs being retained. Admixture analyses were performed using a range of values for the number of estimated ancestry clusters (K = 2–15), to explore the structure in the dataset. To avoid convergence to local optima, the analysis was repeated 100 times, and the replicate with the highest likelihood was chosen.

### Principal components analysis

For the principal components analysis, a variance covariance matrix was computed from the genotype likelihoods of the various samples using *ngsCovar* [51,52]. For this analysis, only polymorphic sites with a minor allele frequency greater than 0.05 were used. Finally, the principal components of the genotype likelihood data were calculated by eigen-decomposition of the variance covariance matrix in *R* (v3.2.1) [53].

### Reconstruction of the phylogeny

For each sample, the consensus sequence was generated in *ANGSD* [17] using the -doFasta 1 option. Regions with missing data were filtered out using *trimal* (v1.4.1) [54] with parameters -gappyout, -resoverlap 0.60 and -seqoverlap 60. The phylogenetic trees for each scaffold were constructed using *FastTree2* (v2.1.10) [55], which uses a generalized time-reversible model for sequence evolution. Only the trees with a minimum of 4 samples were retained to infer the phylogenetic relationship between the samples using *ASTRAL-II* [56] with default parameters.

### Calculation of D-statistics

D-statistics were computed in *ANGSD* [17] using a single randomly sampled allele at each site that was covered by at least one read. Sites with mapping or base quality less than 30 were discarded. The D-statistic was computed for all possible triplets of samples from the data, using an Israeli golden jackal [5] as outgroup, i.e. the tree configuration used to compute the D-statistic was (H1, H2; H3, ‘Golden Jackal’). While golden jackals in Israel have been documented to admix with dogs, grey wolves, and African golden wolves (*Canis anthus*) [57], the specific sample perform well as an outgroup for the configurations tested. Between 0.2–2.1 million sites were used to compute the D statistic, depending on the triplet being used for analysis. Only a subset of the triplets lead to trees that allowed to test hypotheses relating to gene flow between the North American wolves and other canids. Following standard procedure, blocks containing 500 markers each were used to perform the block jackknife [58] procedure to estimate the variance of the statistic.

### Admixture graph fitting using qpGraph

We fitted f-statistics based admixture graphs as implemented in qpGraph from the ADMIXTOOLS package [59] to evaluate the position of the Mexican wolf among Eurasian and American grey wolf diversity. As well as to evaluate the position of red, Eastern timber and Great Lakes wolves among modern coyote and American grey wolf diversity. Specific samples used in the graphs are given in S1 Table. We explain the genomic diversity of red, Eastern timber and Great Lakes wolves as a mix between variation found in coyotes and modern American grey wolves. We considered graphs placing the Mexican wolf as either sister to Eurasian wolves or American wolves under several scenarios of gene flow with various genetic clusters in the graph. We obtained one model with a specific topology, which explained the data well (Fig 3) and present all considered graphs in the supplementary (S11 Fig).

### Migration analyses using Treemix

Specific migration events between populations were estimated using Treemix [60]. As with the D-statistic analyses, informative sites were identified for each sample by randomly sampling one allele at each site, where both nucleotides and reads with quality lower than 30 were excluded. Only sites where 2 different alleles were sampled were retained for the analysis, leading to ~158K-1.938M sites being used, depending on the subset the analysis was performed on. Using these sites and treating each sample as its own initial population, a global tree without any migration edges was constructed. This tree was used as the initial tree for all subsequent Treemix analyses. Treemix graphs with 1–5 migration edges were estimated. For each setting, the best Treemix graph was obtained from 100 replicates.

### Genetic affinity and admixture proportion estimates

Genetic affinity between pairs of samples (X and Y) was estimated by the f3 [61,62] statistic using the triplet (‘Golden Jackal’; X, Y) to assess the shared drift between X and Y from the outgroup ‘Golden Jackal’. The genetic affinity of the samples (X) to the ‘Daneborg’ Greenland wolf and the ‘Mexico’ coyote were contrasted by computing the two f3 statistics—f3(‘Golden Jackal’; X, ‘Daneborg’) and f3(‘Golden Jackal’; X, ‘Mexico’). These were computed by the *threepop* program included as part of the Treemix package [60], using the same set of sites that were used to estimate the Treemix tree. The f4 ratio was used to estimate the amount of coyote and Greenland wolf-like ancestry in all samples included in this study. The program *fourpop*, part of the Treemix package [60], was used to compute two f4 statistics for each sample (X)—f4(‘Daneborg’, X; ‘Eurasia 1’, ‘Golden Jackal’) and f4(X, ‘Mexico’; ‘Eurasia 1’, ‘Golden Jackal’). The proportion of ancestry related to the ‘Daneborg’ Greenland wolf was estimated by computing the ratio f4(‘Daneborg’, X; ‘Eurasia 1’, ‘Golden Jackal’)/f4(‘Daneborg’, ‘Mexico’; ‘Eurasia 1’, ‘Golden Jackal’). Similarly, the proportion of ancestry related to the ‘Mexico’ coyote in sample X was computed using the ratio f4(X, ‘Mexico’; ‘Eurasia 1’, ‘Golden Jackal’)/f4(‘Daneborg’, ‘Mexico’; ‘Eurasia 1’, ‘Golden Jackal’). Further details on the f4 ratio and its use in estimating the admixture proportions can be found in Patterson et al. [61].

### Heterozygosity, inbreeding and runs of homozygosity (ROH)

For each sample, the heterozygosity was computed using *ANGSD* [17] under a probabilistic framework based on genotype likelihoods. Reads with mapping quality lower than 20, and bases with base qualities less than 20, were excluded from the analyses. The heterozygosity and its variance were calculated from 100 sets of variant sites obtained by bootstrapping on the polymorphic sites. The inbreeding coefficient for each sample was estimated under a probabilistic framework using *ngsF* [63], which allows for estimation of inbreeding coefficients without calling genotypes. The genotype likelihoods dataset that was previously calculated for the *NGSadmix* [18] analysis, was used for computing inbreeding. To avoid convergence to local maxima, the approximated-EM algorithm was started 20 times from random initial values, and the run with the highest likelihood was used as starting values for the final EM run. We selected 7 wolf samples—Mexico 1, IRNP, Banks Island, North Baffin, Daneborg, Pacific Coast and Yellowstone 2—for the ROH analysis since they spanned all the interesting wolf clades, and had a minimum genome coverage of 10x (except IRNP, which has a genome coverage of 9x). Genotype calling was performed using GATK (v3.3.0) [49] haplotype caller, restricting the analysis to only variable sites identified in the full set of samples (~ 10.5 million variable sites). Subsequently, we identified ROH using plink (v1.9) [64], only allowing regions longer than 1 Mb, with a minimum of 100 SNPs.

## Supporting information

S1 Fig Estimated error rates. The estimated base-specific and individual wide error rates are shown for all samples, using the “Daneborg” Polar wolf as the reference sample and the golden jackal as the outgroup. Individuals are represented by different colours. The individual wide error rates are shown on the right. Numerical values for all samples are given in supplementary S1 Table.
(DOCX)

S2 Fig Admixture plots for K = 2–15. The admixture proportions are shown for a range of estimated ancestry clusters (K = 2–15). Each row corresponds to a specific value of K, while each sample is represented by a column. The colours represent ancestry clusters, while the main groups of samples are separated by solid lines while subpopulations are demarcated using dotted lines. The clusters are consistent through the different values of K, except for the lime green colour at the K = 14, where it represents a cluster of coyotes which disappears at K = 15. This might be due to convergence to different local optima. In general, admixture analyses with high number of clusters must be interpreted with care due to the large number of parameters being estimated.
(DOCX)

S3 Fig Phylogeny estimated using ASTRAL-II. **A.** The relationship between the different samples, estimated as a bifurcating tree in Astral. The branch lengths are represented in coalescent time units. Therefore, the terminal (leaf nodes) branch lengths are arbitrarily scaled. The local posterior probability for each node is given instead of a bootstrap value. **B.** The Astral phylogeny represented using collapsed populations, where each node represents a monophyletic group from the tree shown in A. The only population/group which showed non-monophyly in the phylogeny in A was the Eastern timber/Great Lakes wolves, which were split into 3 different groups. The group Eastern timber/Great Lakes wolf 1 includes the samples Algonquin 2 and Algonquin 3, the group Eastern timber/Great Lakes wolf 2 include the samples Algonquin 1 and the grey wolf from Isle Royale National Park, and finally the last group, Eastern timber/Great Lakes wolf 3 contains one sample, the Great Lakes wolf from Minnesota. **C.** The bar charts show the different frequencies of the three possible bipartitions obtained from an unrooted tree at many of the labelled branches in the Astral phylogeny shown in B. The red bar represents the topology shown in the tree, while the two blue bars represent the two other alternative topologies. The dotted line shows the frequency 0.33—previous theoretical work (1) has shown that the frequency of the true topology must be at least 0.33. (1. Allman ES, Degnan JH, Rhodes JA. Identifying the rooted species tree from the distribution of unrooted gene trees under the coalescent. J Math Biol. 2011 Jun 1;62(6):833–62.)
(DOCX)

S4 Fig Principal components analysis for all samples in the study. The first 4 principal components, estimated from the genotype likelihood data, are plotted in the two panels. All the individuals are included in this analysis. Different populations are indicated using different colours. Circles indicate samples sequenced as part of this study, while squares represent previously published samples.
(DOCX)

S5 Fig Principal components analysis for red wolves and coyotes. The first 4 principal components, estimated from the genotype likelihoods, are plotted in the two panels. Only coyotes and red wolves are included in this analysis. Different populations are shown using different colours. Circles indicate samples sequenced as part of this study, while squares represent previously published samples.
(DOCX)

S6 Fig D-statistics for the tree configuration (H1, Daneborg Polar Wolf (GW); Mexico coyote (MC), Golden Jackal (GJ)). This figure shows the D-statistic (ABBA-BABA test) using the Golden Jackal as the outgroup. The error bars indicate 1 and 3 standard errors of the D-statistic. Different canines were used as part of the ingroup (H1), along with the “Daneborg” Polar wolf (H2). The yellow line indicates the null expectation in the absence of gene flow from any of the ingroup samples to the MC (D = 0). A significantly positive test statistic implies higher gene flow between GW-MC than H1-MC, while a negative test statistic implies higher gene flow between H1-MC than GW-MC. Note the positive test statistic for the Eurasian wolves (Eurasia 1–3) is likely a result of some gene flow between them and the outgroup GJ.
(DOCX)

S7 Fig D-statistics for the tree configuration (H1, Daneborg Polar Wolf (GW); Eurasia 2 (EW2), Golden Jackal (GJ)). This figure shows the D-statistic (ABBA-BABA test) using the golden jackal as the outgroup. The error bars indicate 1 and 3 standard errors of the D-statistic. Different canines were used as part of the ingroup (H1), along with the “Daneborg” Polar wolf. The yellow line indicates the null expectation in the absence of gene flow from any of the ingroup samples to the EW2 (D = 0). A significantly positive test statistic implies higher gene flow between GW-EW2 than H1-EW2, while a negative test statistic implies higher gene flow between H1-EW2 than GW-EW2. The significantly positive D-statistic values for many of the samples including the red wolves, Eastern timber/Great Lakes wolves and the Mexican wolves can be attributed to outgroup attraction due to gene flow into these samples from coyotes. Outside of the Eurasian wolves, the only samples showing any evidence of gene flow from the Eurasia 2 are the Alaskan wolves, Alaska 1 and Alaska 2.
(DOCX)

S8 Fig Treemix analysis of 39 samples in the study. Treemix analysis for all samples in the data set except the Polar wolf, “Krummelangsø”. The graphs estimated by Treemix with 0–4 migration edges are shown in panels A-E. The respective residuals and log-likelihoods are also show alongside the estimated graphs. The colour of migration edges corresponds to migration weight indicated by the colour scale bar to the left. The long drift lengths of some of these branches, e.g. “Yellowstone 1”, the “Alabama” coyote, “Red wolf 2”, can be explained by higher estimated error rates in these samples.
(DOCX)

S9 Fig Treemix of wolves. Treemix analysis for all grey wolves in the data set except the Polar wolf, “Krummelangsø”. The log-likelihood showed that adding migration edges to the maximum likelihood tree did not result in a significant improvement to the fit of the data; therefore, only the maximum likelihood, that is, the tree with no migration edges, is shown here. The long drift branch of the “Yellowstone 1” wolf can be attributed to high estimated error rates in this sample.
(DOCX)

S10 Fig Treemix analysis of coyotes and wolf like canids. Treemix analysis for all the non-North American grey wolves in the dataset. For orienting this graph, we did include 6 wolves, viz., the Eurasian wolves, “Yellowstone 2”, “Daneborg” Polar wolf and “Mexico 1”. The panels A-D includes 0–3 migration edges, where the colour of migration edges corresponds to migration weight, shown by the colour bar scale to the right.
(DOCX)

S11 Fig QP-admixture graph´s of Mexican wolf. Various admixture graphs for the formation of Mexican wolves, the specific samples used in each cluster are given in supplementary S1 Table. Internal nodes denoted by letters from a to m are hypothesised meta-populations. Tip nodes indicate the sampled genomes used to fit the graph. Dotted connecting lines represent admixture events, with the percentages indicating the admixture proportions. Solid connecting lines represent the divergence between populations with the numbers indicating their corresponding branch lengths.
(DOCX)

S12 Fig Genetic affinity to the Mexico coyote. The genetic affinity of the North American canines, plotted on a map. Circles represent grey wolves and squares indicate wolf like canines. Colours represent genetic affinity, computed using the f3 statistic with the golden jackal as the outgroup. The more closely related a sample is to the “Mexico” coyote, the deeper red its symbol. The colour bar scale on the right shows the scale of the f3 statistic.
(DOCX)

S13 Fig Runs of homozygosity for selected wolves. Percentage of genome contained in runs of homozygosity (ROH). Only regions longer than 1Mb and containing a minimum of 100 SNPs were considered to be ROH. Only a representative set of 7 wolves, with greater than 10x genome coverage were used in this analysis. The results shows that the Mexican wolf—Mexico 1—has the highest fraction of the genome in ROHs, followed by the IRNP wolf, and the Greenland wolf—Daneborg.
(DOCX)

S1 Table Sample information. (PDF)
