# Supplementary figures and images for: Population genomics of grey wolves and wolf-like canids in North America
Source: PLoS Genet. 2018 Nov 12;14(11):e1007745. doi: 10.1371/journal.pgen.1007745 (PMC6231604; doi:10.1371/journal.pgen.1007745)

**Figure S1: Estimated error rates.**


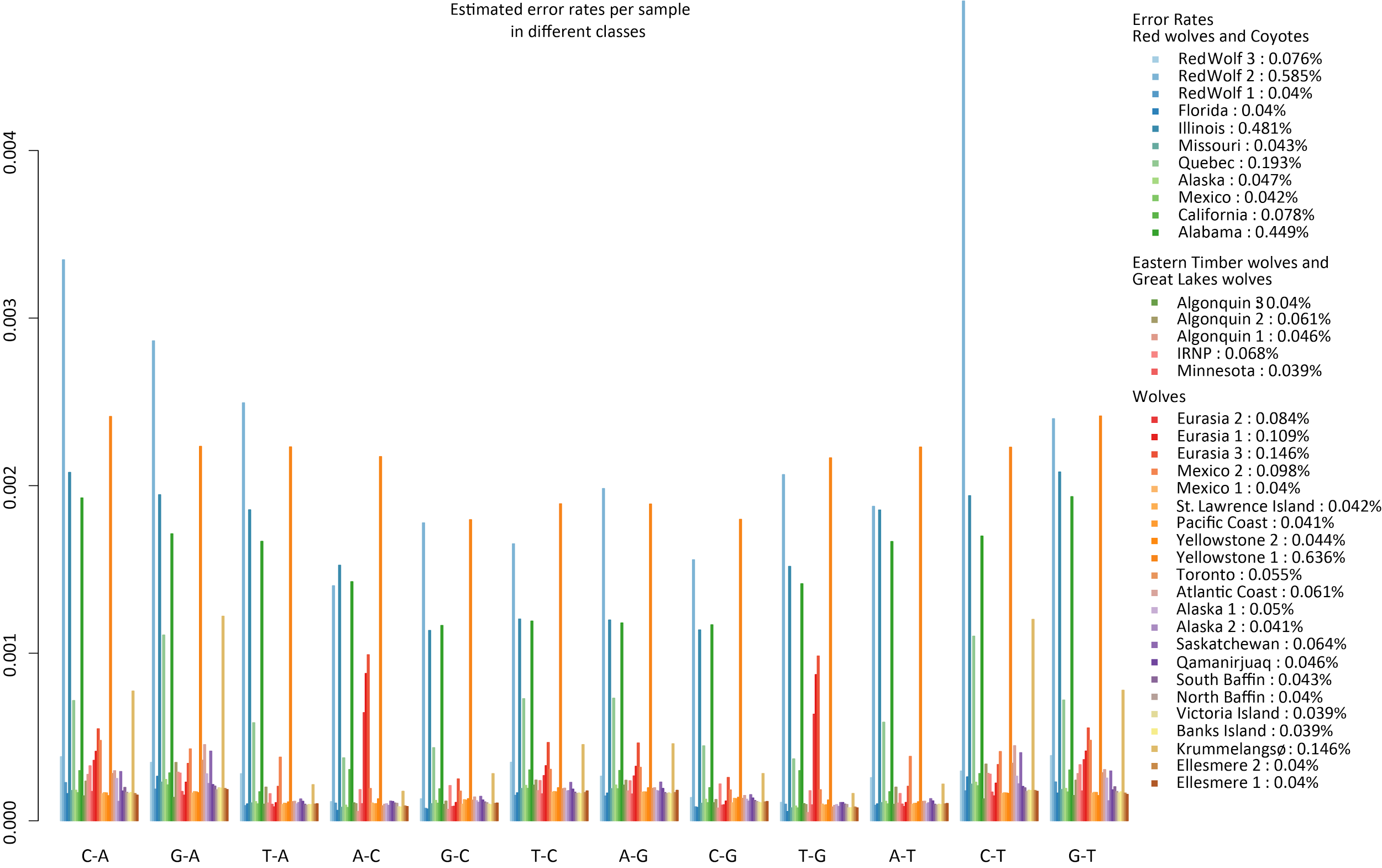

Supplement: S1 Fig — The estimated base-specific and individual wide error rates are shown for all samples, using the “Daneborg” Polar wolf as the reference sample and the golden jackal as the outgroup. Individuals are represented by different colours. The individual wide error rates are shown on the right. Numerical values for all samples are given in supplementary S1 Table. (DOCX) [file pgen.1007745.s001.docx]

**Figure S2. Admixture plots for K=2-15.**


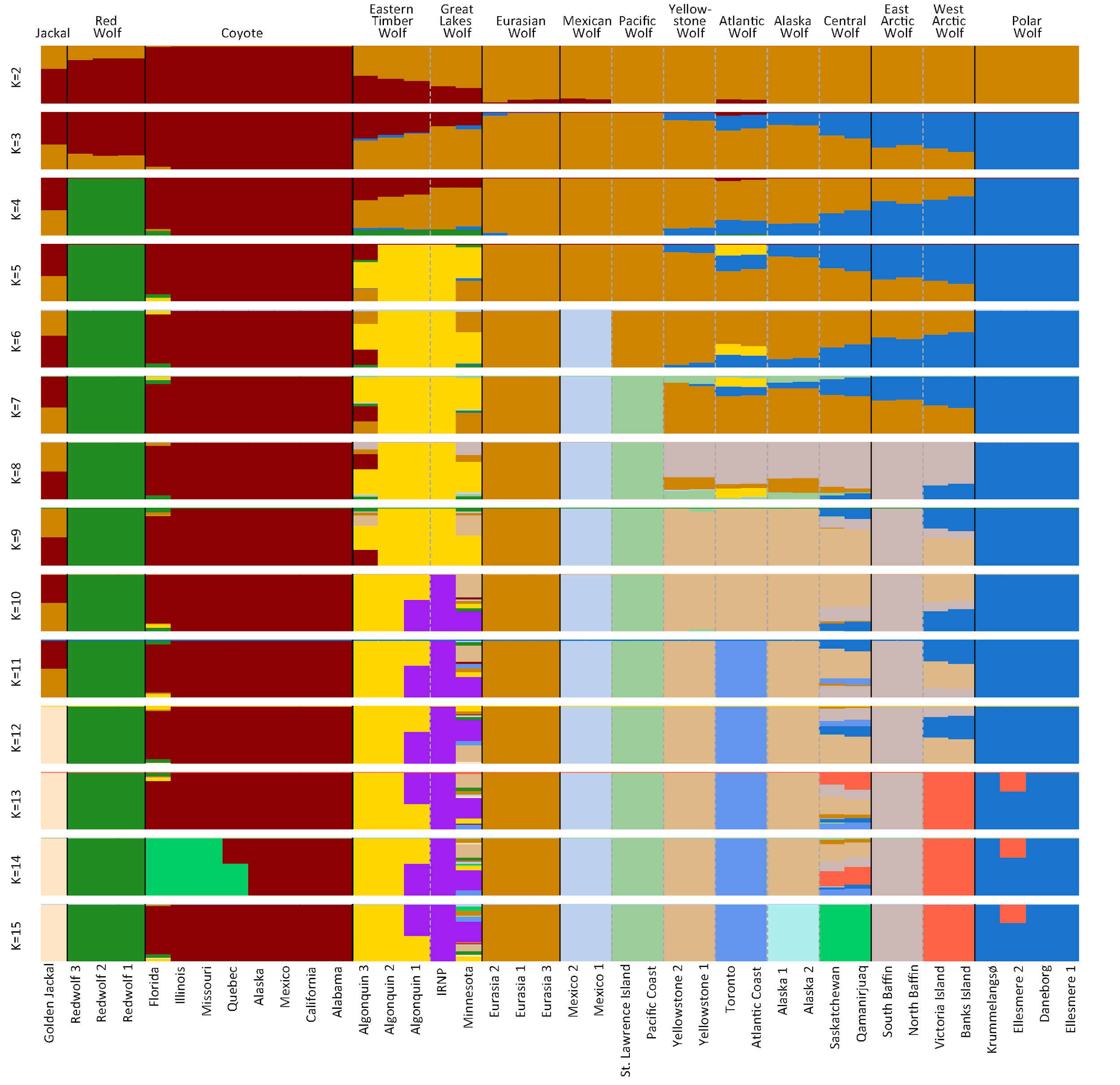

Supplement: S2 Fig — The admixture proportions are shown for a range of estimated ancestry clusters (K = 2–15). Each row corresponds to a specific value of K, while each sample is represented by a column. The colours represent ancestry clusters, while the main groups of samples are separated by solid lines while subpopulations are demarcated using dotted lines. The clusters are consistent through the different values of K, except for the lime green colour at the K = 14, where it represents a cluster of coyotes which disappears at K = 15. This might be due to convergence to different local optima. In general, admixture analyses with high number of clusters must be interpreted with care due to the large number of parameters being estimated. (DOCX) [file pgen.1007745.s002.docx]

**Figure S3: Astral phylogeny.**


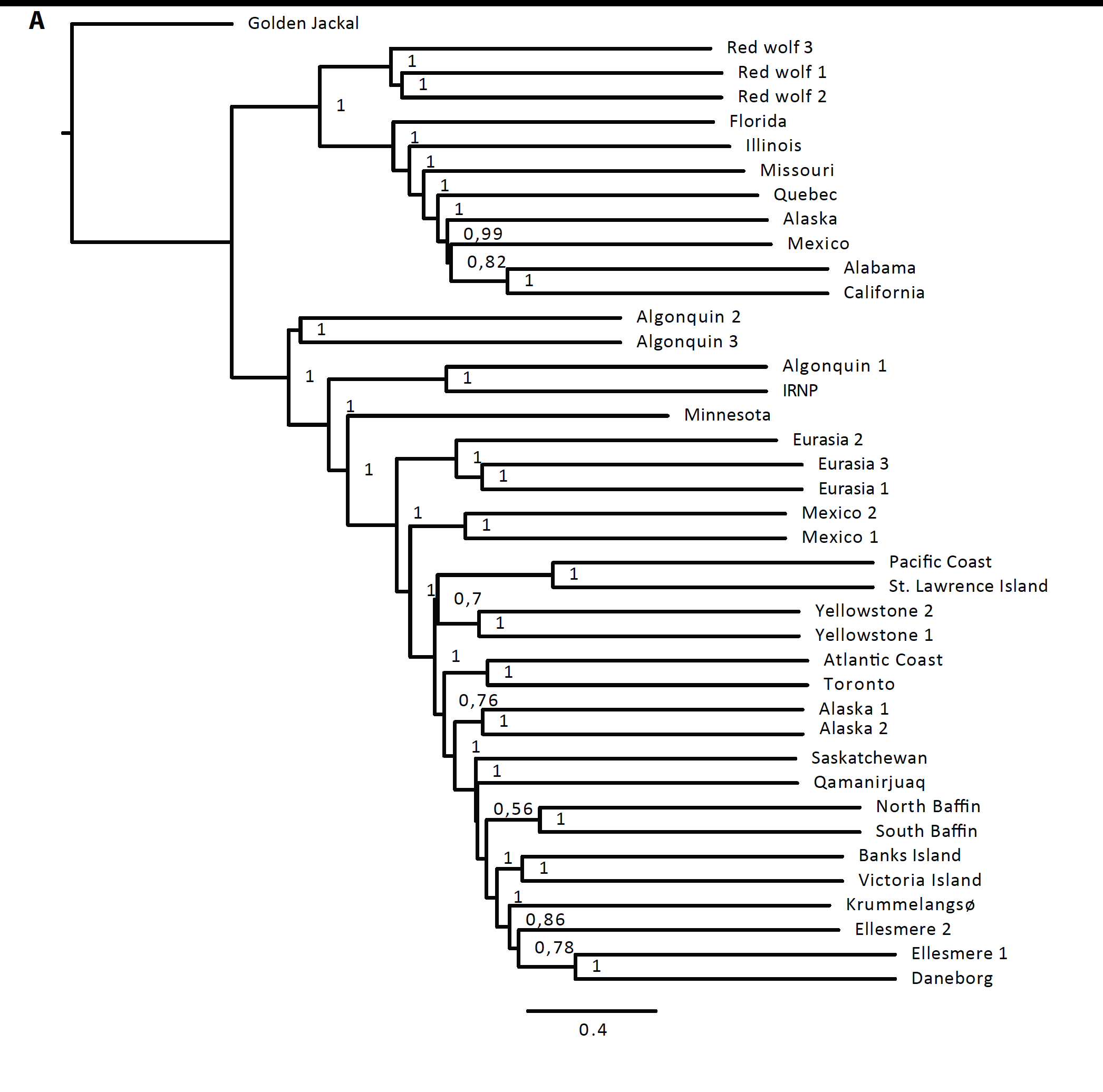


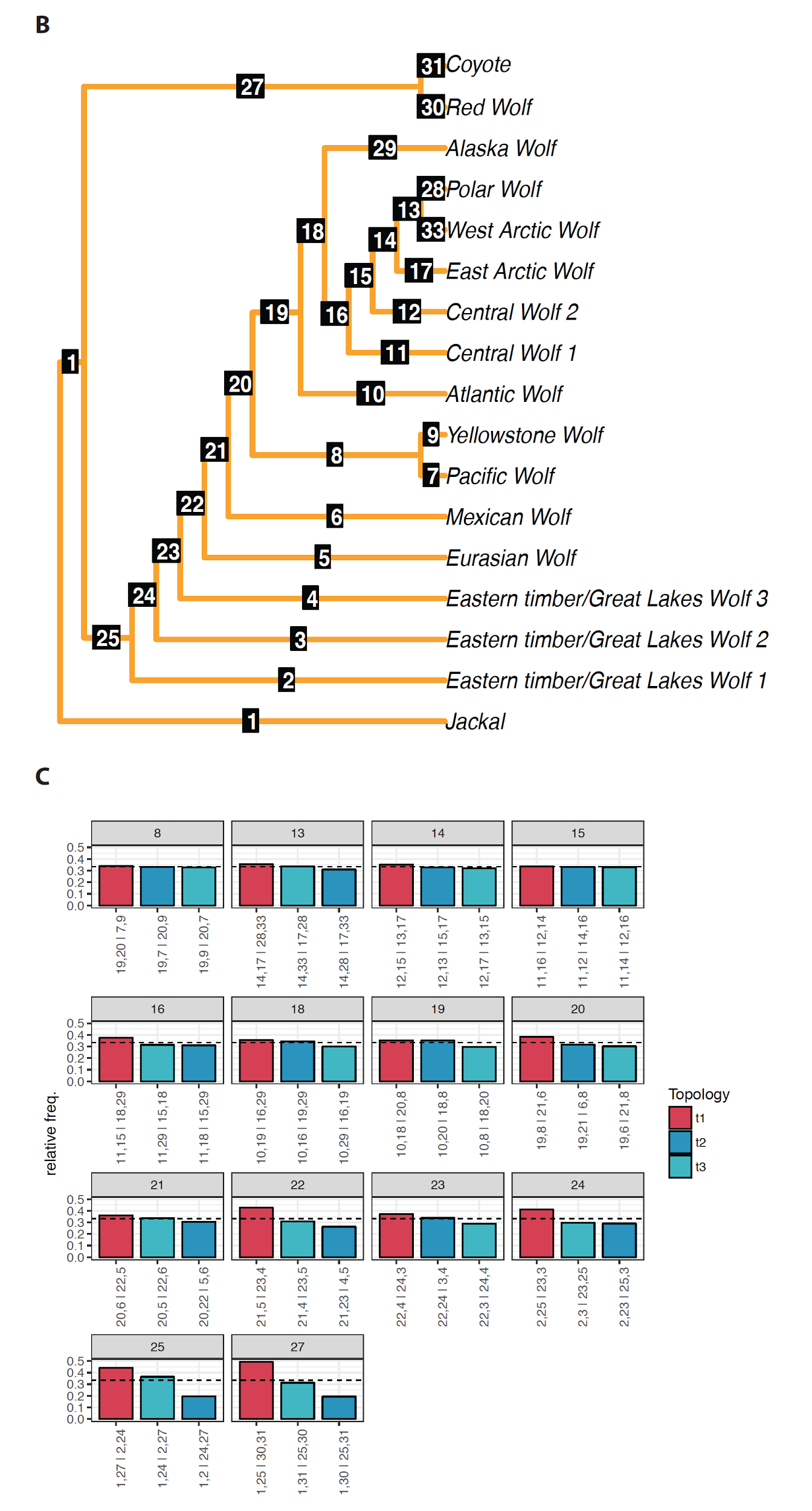

Supplement: S3 Fig — A. The relationship between the different samples, estimated as a bifurcating tree in Astral. The branch lengths are represented in coalescent time units. Therefore, the terminal (leaf nodes) branch lengths are arbitrarily scaled. The local posterior probability for each node is given instead of a bootstrap value. B. The Astral phylogeny represented using collapsed populations, where each node represents a monophyletic group from the tree shown in A. The only population/group which showed non-monophyly in the phylogeny in A was the Eastern timber/Great Lakes wolves, which were split into 3 different groups. The group Eastern timber/Great Lakes wolf 1 includes the samples Algonquin 2 and Algonquin 3, the group Eastern timber/Great Lakes wolf 2 include the samples Algonquin 1 and the grey wolf from Isle Royale National Park, and finally the last group, Eastern timber/Great Lakes wolf 3 contains one sample, the Great Lakes wolf from Minnesota. C. The bar charts show the different frequencies of the three possible bipartitions obtained from an unrooted tree at many of the labelled branches in the Astral phylogeny shown in B. The red bar represents the topology shown in the tree, while the two blue bars represent the two other alternative topologies. The dotted line shows the frequency 0.33—previous theoretical work (1) has shown that the frequency of the true topology must be at least 0.33. (1. Allman ES, Degnan JH, Rhodes JA. Identifying the rooted species tree from the distribution of unrooted gene trees under the coalescent. J Math Biol. 2011 Jun 1;62(6):833–62.) (DOCX) [file pgen.1007745.s003.docx]

**Figure S4: Principal components analysis for all samples in the study.**


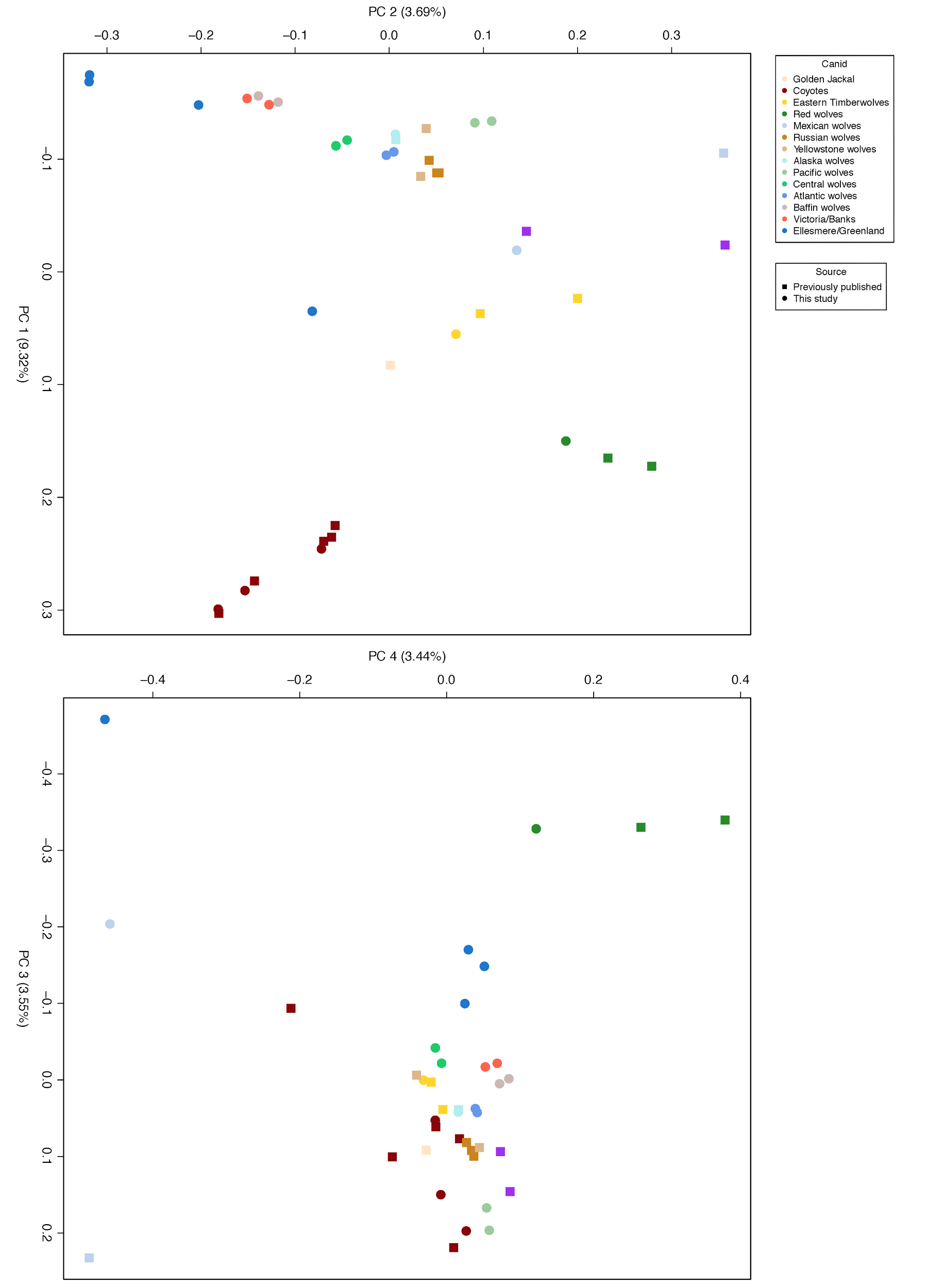

Supplement: S4 Fig — The first 4 principal components, estimated from the genotype likelihood data, are plotted in the two panels. All the individuals are included in this analysis. Different populations are indicated using different colours. Circles indicate samples sequenced as part of this study, while squares represent previously published samples. (DOCX) [file pgen.1007745.s004.docx]

**Figure S5: Principal components analysis for red wolves and coyotes.**


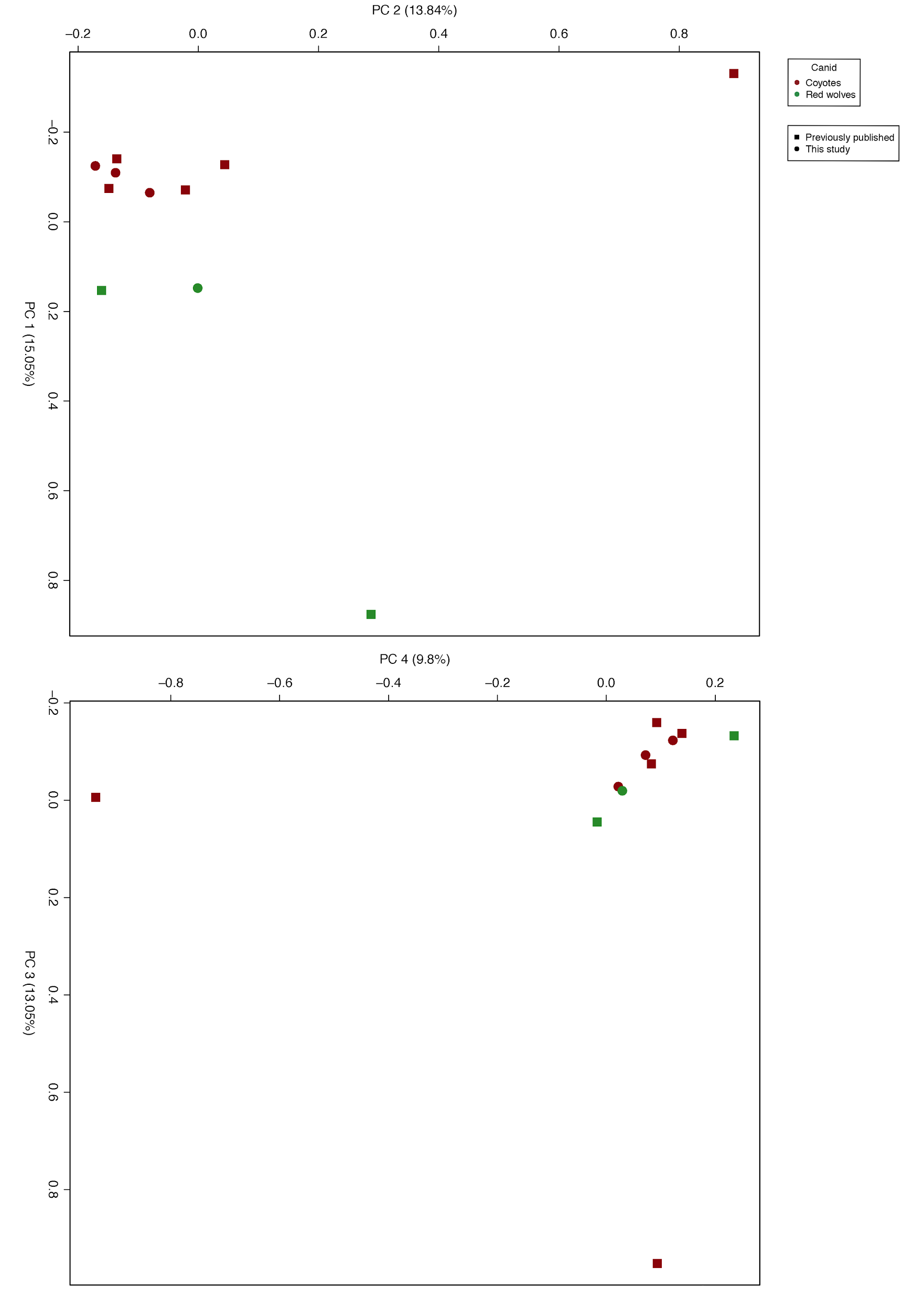

Supplement: S5 Fig — The first 4 principal components, estimated from the genotype likelihoods, are plotted in the two panels. Only coyotes and red wolves are included in this analysis. Different populations are shown using different colours. Circles indicate samples sequenced as part of this study, while squares represent previously published samples. (DOCX) [file pgen.1007745.s005.docx]

**Figure S8: Treemix analysis of 39 samples in the study.**


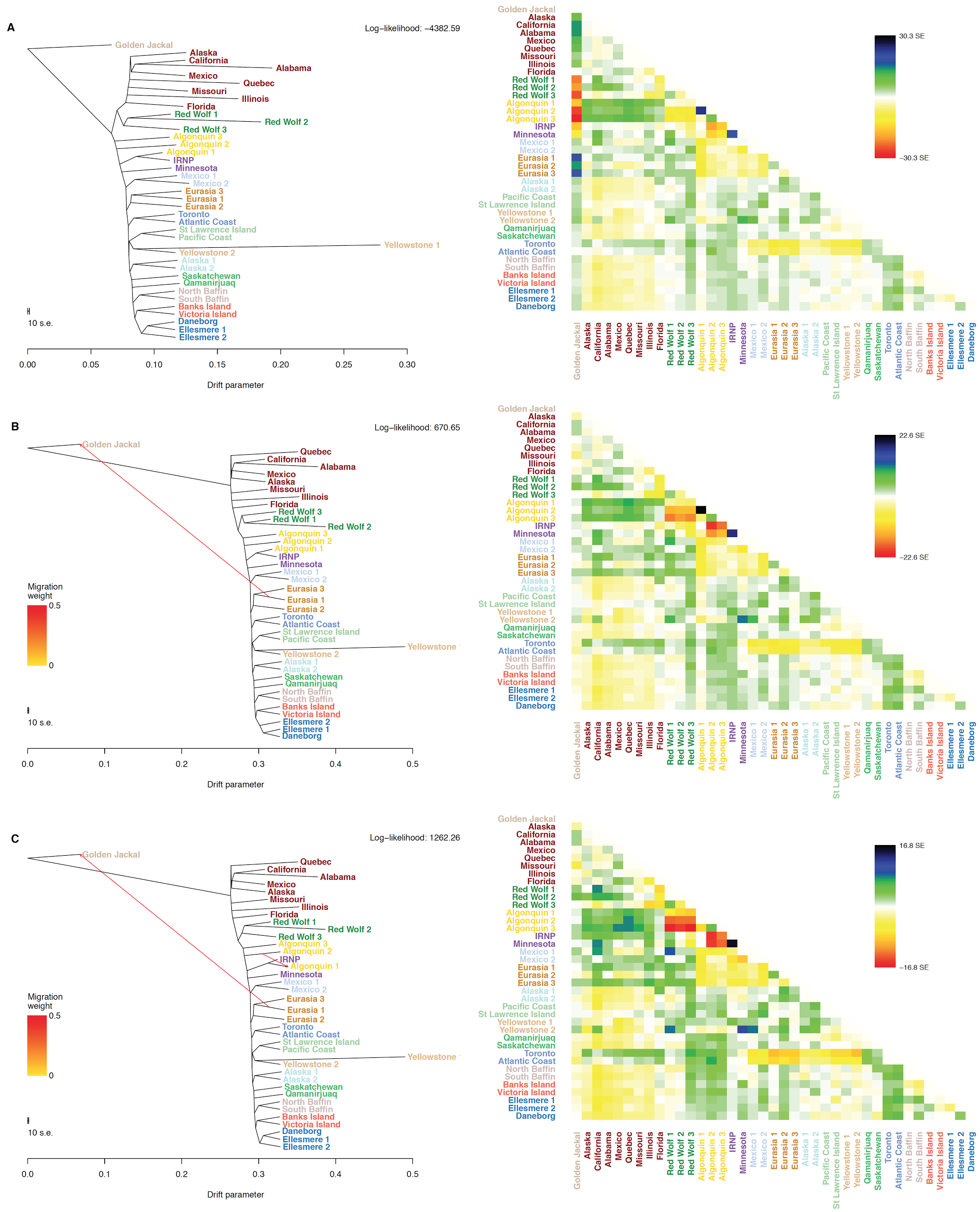


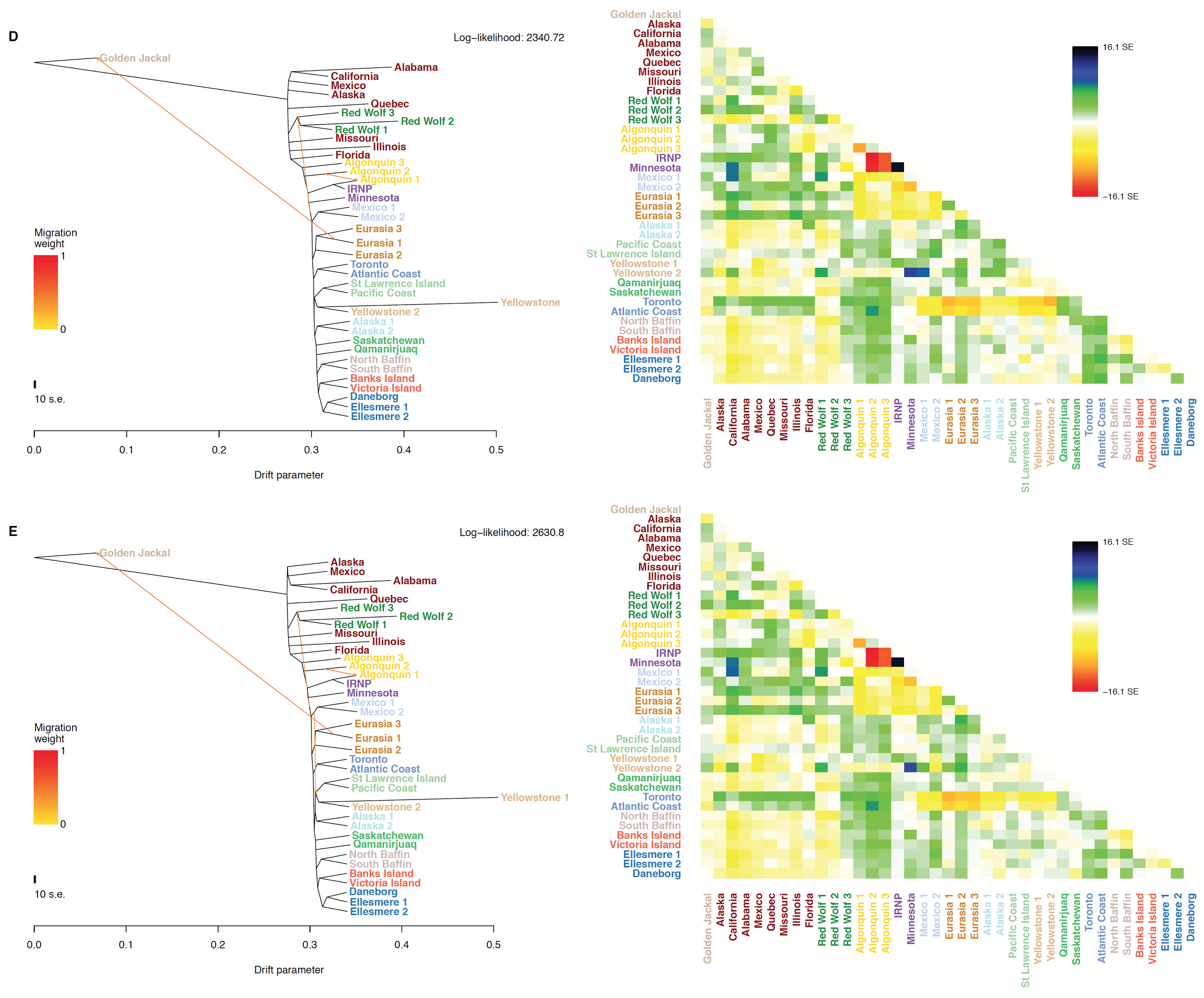

Supplement: S8 Fig — Treemix analysis for all samples in the data set except the Polar wolf, “Krummelangsø”. The graphs estimated by Treemix with 0–4 migration edges are shown in panels A-E. The respective residuals and log-likelihoods are also show alongside the estimated graphs. The colour of migration edges corresponds to migration weight indicated by the colour scale bar to the left. The long drift lengths of some of these branches, e.g. “Yellowstone 1”, the “Alabama” coyote, “Red wolf 2”, can be explained by higher estimated error rates in these samples. (DOCX) [file pgen.1007745.s008.docx]

**Figure S9: Treemix of wolves.**


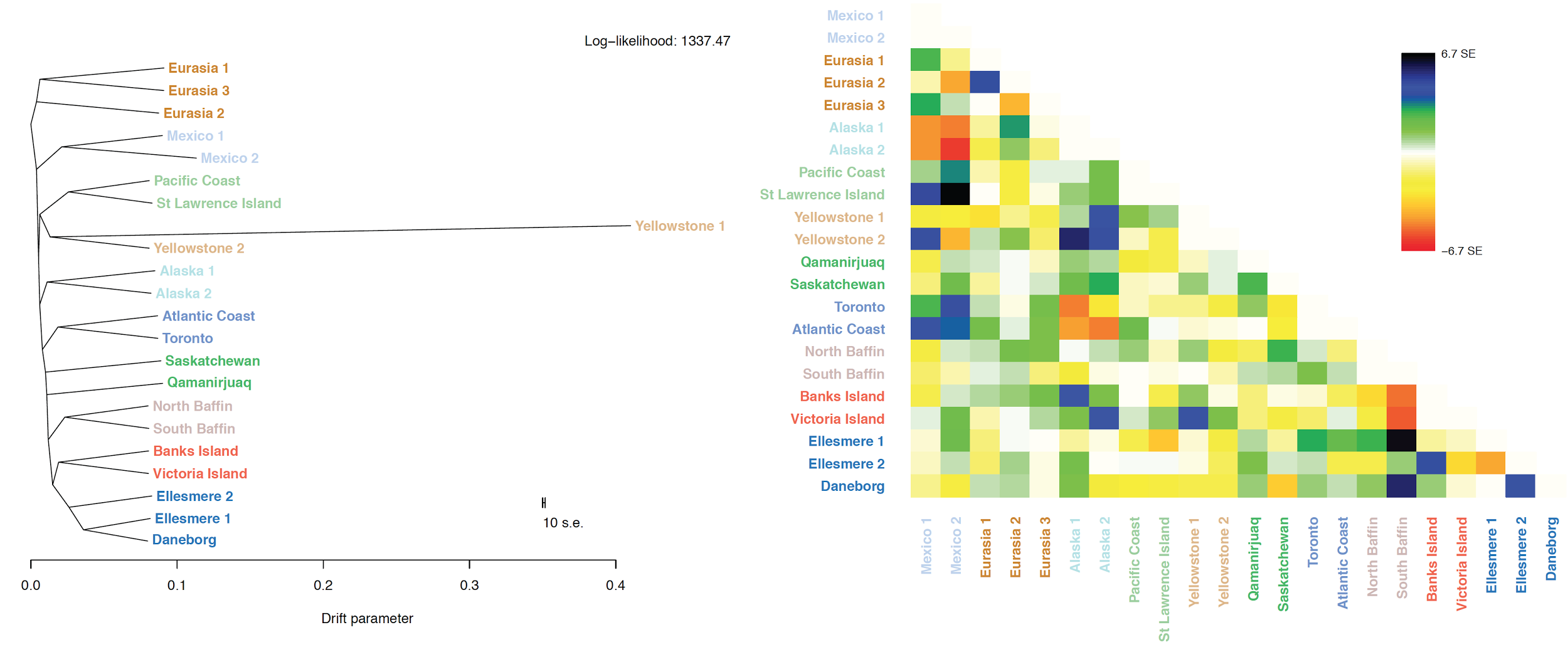

Supplement: S9 Fig — Treemix analysis for all grey wolves in the data set except the Polar wolf, “Krummelangsø”. The log-likelihood showed that adding migration edges to the maximum likelihood tree did not result in a significant improvement to the fit of the data; therefore, only the maximum likelihood, that is, the tree with no migration edges, is shown here. The long drift branch of the “Yellowstone 1” wolf can be attributed to high estimated error rates in this sample. (DOCX) [file pgen.1007745.s009.docx]

**Figure S10: Treemix analysis of coyotes and wolf like canids.**


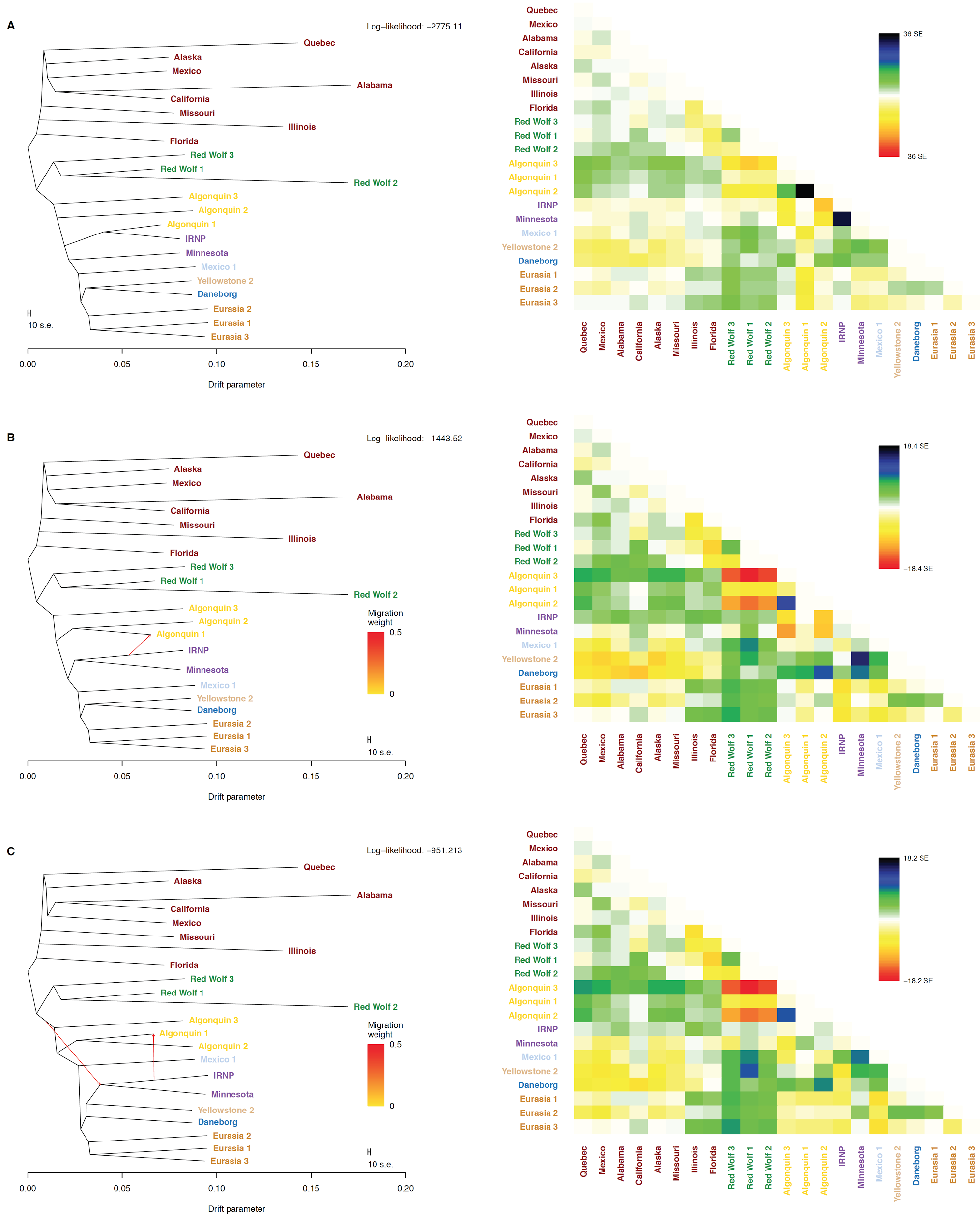


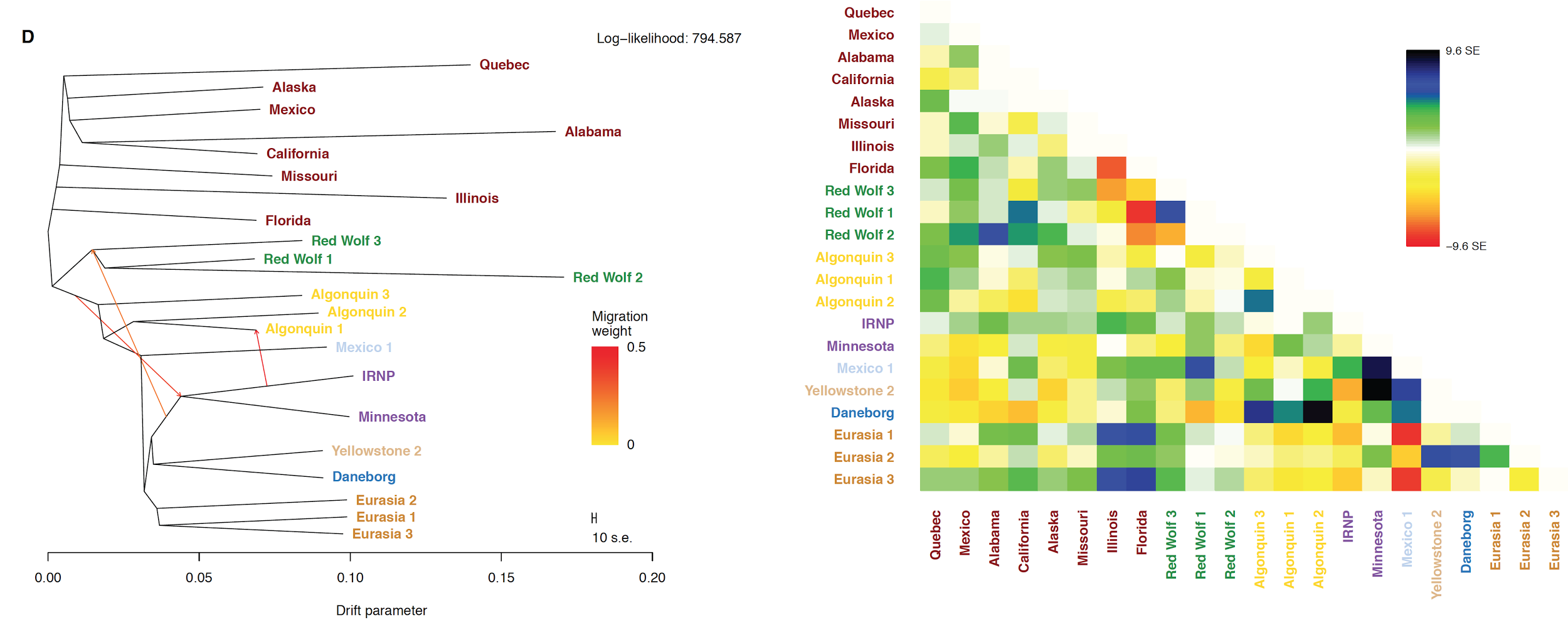

Supplement: S10 Fig — Treemix analysis for all the non-North American grey wolves in the dataset. For orienting this graph, we did include 6 wolves, viz., the Eurasian wolves, “Yellowstone 2”, “Daneborg” Polar wolf and “Mexico 1”. The panels A-D includes 0–3 migration edges, where the colour of migration edges corresponds to migration weight, shown by the colour bar scale to the right. (DOCX) [file pgen.1007745.s010.docx]

**Figure S11: QP-admixture graph´s of Mexican wolf.**


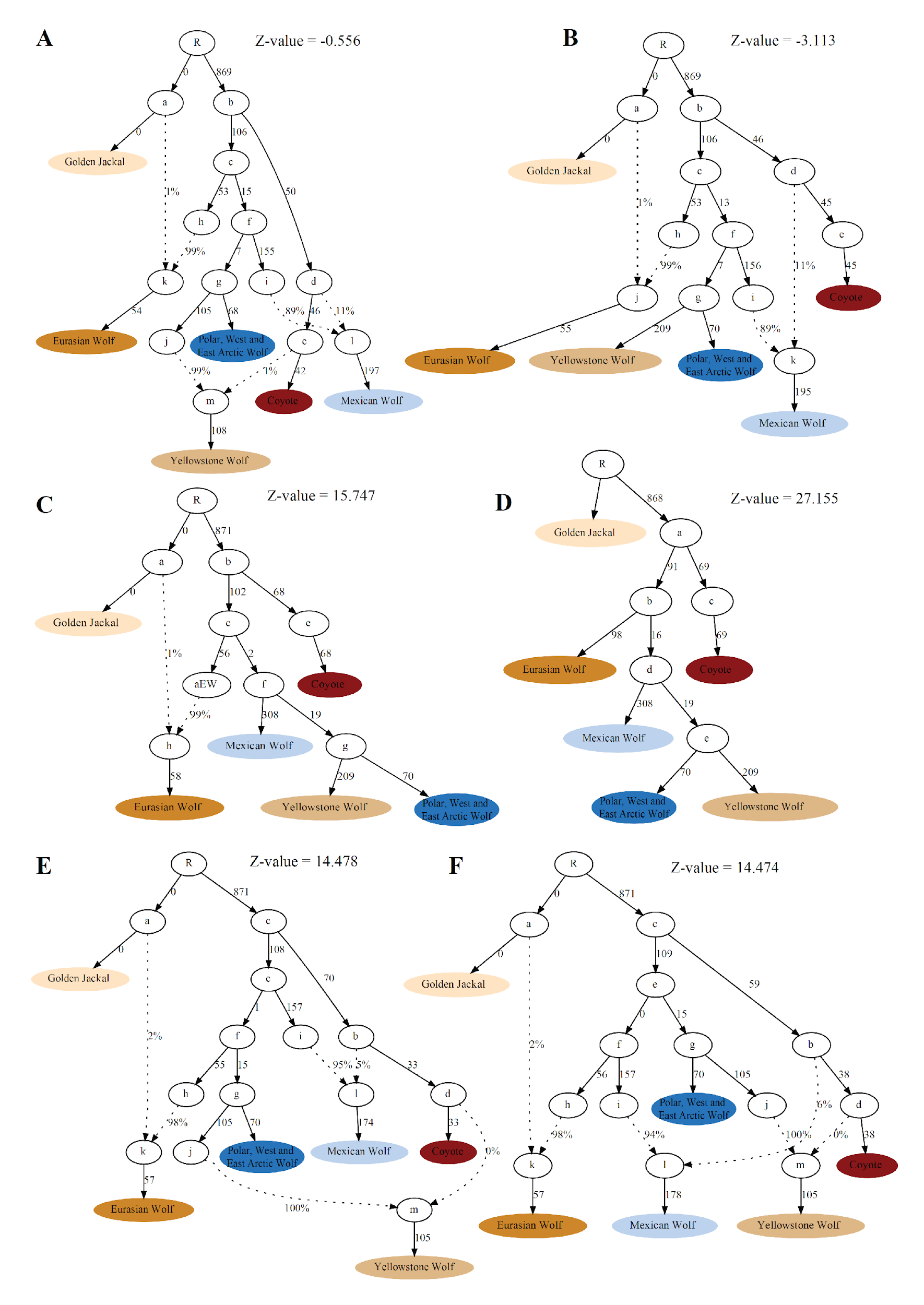

Supplement: S11 Fig — Various admixture graphs for the formation of Mexican wolves, the specific samples used in each cluster are given in supplementary S1 Table. Internal nodes denoted by letters from a to m are hypothesised meta-populations. Tip nodes indicate the sampled genomes used to fit the graph. Dotted connecting lines represent admixture events, with the percentages indicating the admixture proportions. Solid connecting lines represent the divergence between populations with the numbers indicating their corresponding branch lengths. (DOCX) [file pgen.1007745.s011.docx]

**Figure S12: Genetic affinity to the Mexico coyote.**


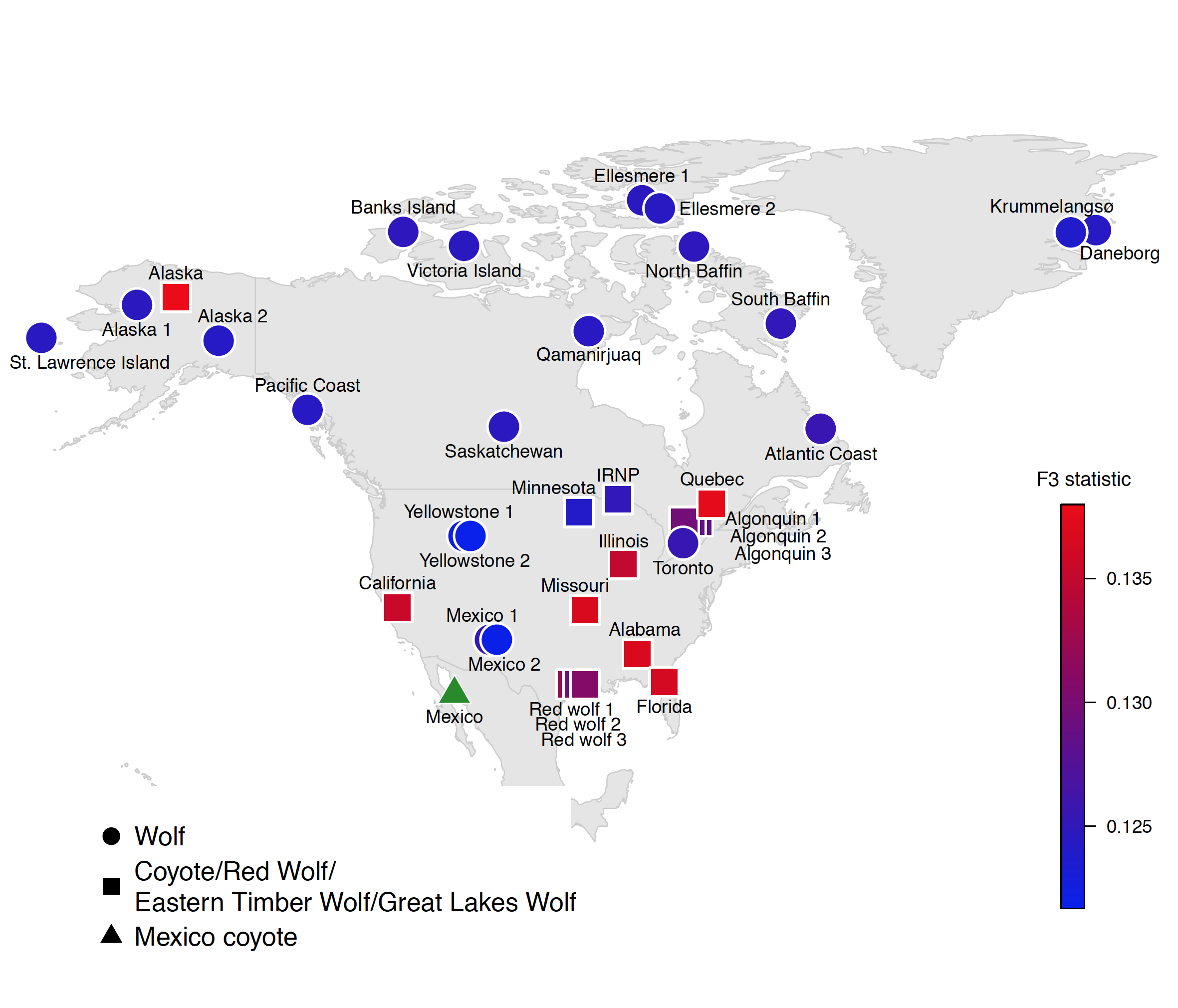

Supplement: S12 Fig — The genetic affinity of the North American canines, plotted on a map. Circles represent grey wolves and squares indicate wolf like canines. Colours represent genetic affinity, computed using the f3 statistic with the golden jackal as the outgroup. The more closely related a sample is to the “Mexico” coyote, the deeper red its symbol. The colour bar scale on the right shows the scale of the f3 statistic. (DOCX) [file pgen.1007745.s012.docx]

**Figure S13: Runs of homozygosity for selected wolves**


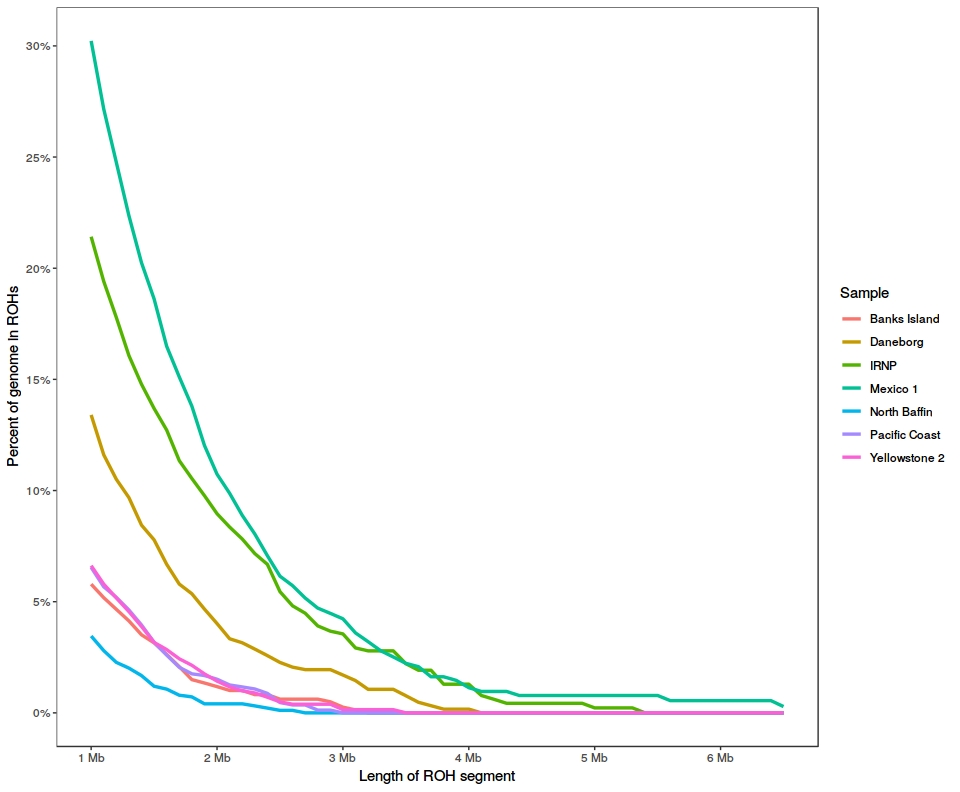

Supplement: S13 Fig — Percentage of genome contained in runs of homozygosity (ROH). Only regions longer than 1Mb and containing a minimum of 100 SNPs were considered to be ROH. Only a representative set of 7 wolves, with greater than 10x genome coverage were used in this analysis. The results shows that the Mexican wolf—Mexico 1—has the highest fraction of the genome in ROHs, followed by the IRNP wolf, and the Greenland wolf—Daneborg. (DOCX) [file pgen.1007745.s013.docx]
