# Supplementary material for: Population genomics of grey wolves and wolf-like canids in North America
Source: PLoS Genet. 2018 Nov 12;14(11):e1007745. doi: 10.1371/journal.pgen.1007745 (PMC6231604; doi:10.1371/journal.pgen.1007745)

**Figure S6: D-statistics for the tree configuration (H1, Daneborg Polar Wolf (GW); Mexico coyote (MC), Golden Jackal (GJ)).**


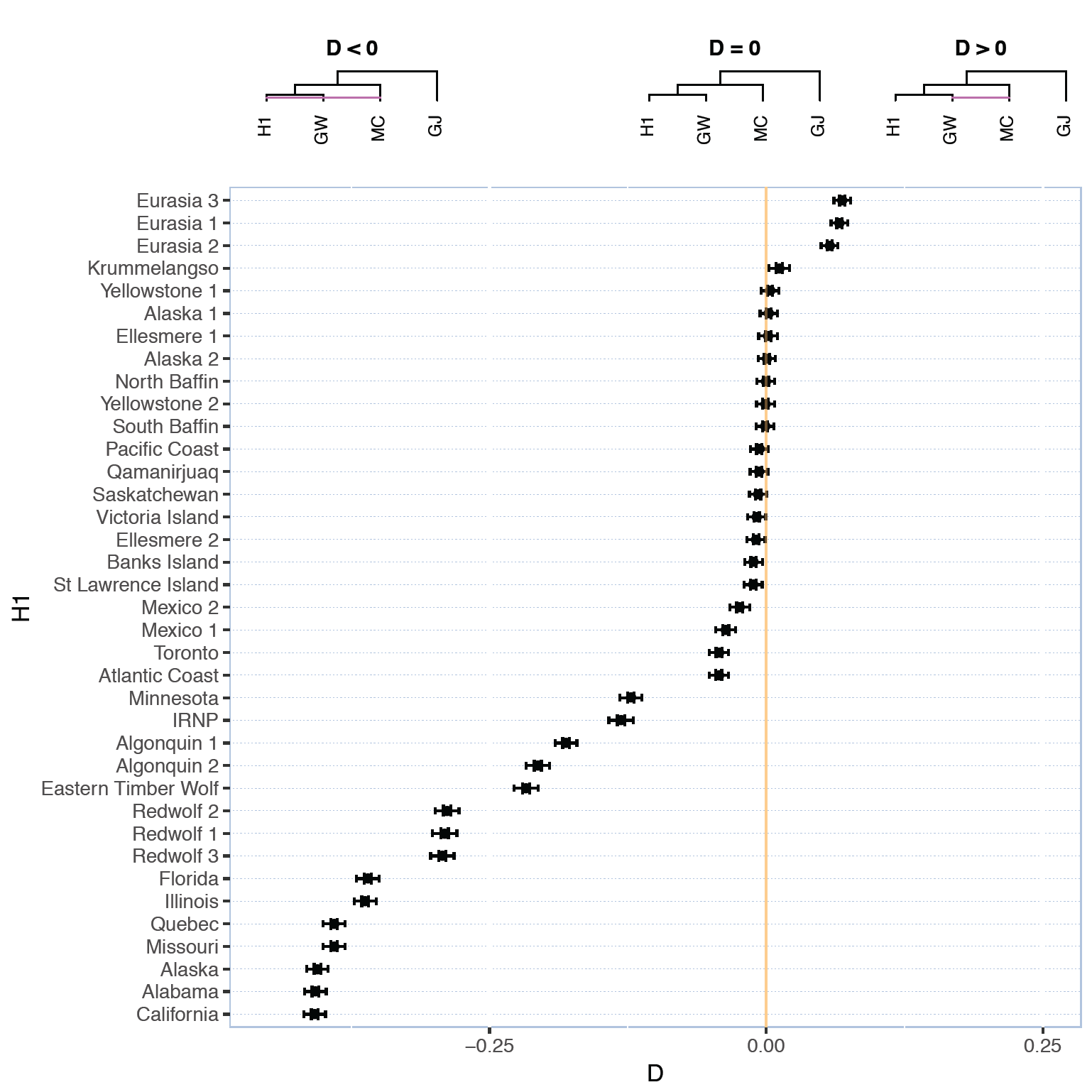

Supplement: S6 Fig — This figure shows the D-statistic (ABBA-BABA test) using the Golden Jackal as the outgroup. The error bars indicate 1 and 3 standard errors of the D-statistic. Different canines were used as part of the ingroup (H1), along with the “Daneborg” Polar wolf (H2). The yellow line indicates the null expectation in the absence of gene flow from any of the ingroup samples to the MC (D = 0). A significantly positive test statistic implies higher gene flow between GW-MC than H1-MC, while a negative test statistic implies higher gene flow between H1-MC than GW-MC. Note the positive test statistic for the Eurasian wolves (Eurasia 1–3) is likely a result of some gene flow between them and the outgroup GJ. (DOCX) [file pgen.1007745.s006.docx]
