# Supplementary material for: Population genomics of grey wolves and wolf-like canids in North America
Source: PLoS Genet. 2018 Nov 12;14(11):e1007745. doi: 10.1371/journal.pgen.1007745 (PMC6231604; doi:10.1371/journal.pgen.1007745)

**Figure S7: D-statistics for the tree configuration (H1, Daneborg Polar Wolf (GW); Eurasia 2 (EW2), Golden Jackal (GJ)).**


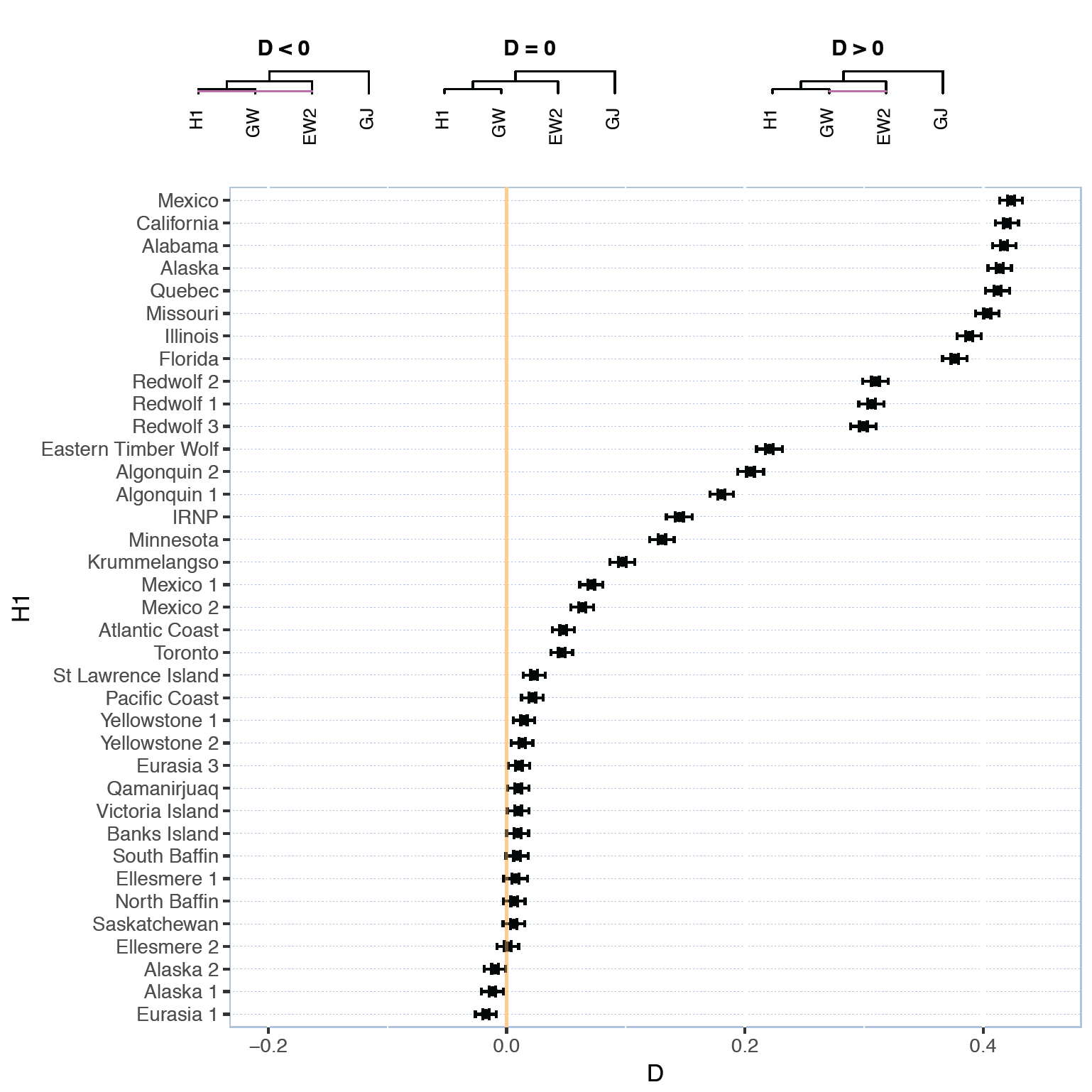

Supplement: S7 Fig — This figure shows the D-statistic (ABBA-BABA test) using the golden jackal as the outgroup. The error bars indicate 1 and 3 standard errors of the D-statistic. Different canines were used as part of the ingroup (H1), along with the “Daneborg” Polar wolf. The yellow line indicates the null expectation in the absence of gene flow from any of the ingroup samples to the EW2 (D = 0). A significantly positive test statistic implies higher gene flow between GW-EW2 than H1-EW2, while a negative test statistic implies higher gene flow between H1-EW2 than GW-EW2. The significantly positive D-statistic values for many of the samples including the red wolves, Eastern timber/Great Lakes wolves and the Mexican wolves can be attributed to outgroup attraction due to gene flow into these samples from coyotes. Outside of the Eurasian wolves, the only samples showing any evidence of gene flow from the Eurasia 2 are the Alaskan wolves, Alaska 1 and Alaska 2. (DOCX) [file pgen.1007745.s007.docx]
