# Supplementary material for: Population genomics of grey wolves and wolf-like canids in North America
Source: PLoS Genet. 2018 Nov 12;14(11):e1007745. doi: 10.1371/journal.pgen.1007745 (PMC6231604; doi:10.1371/journal.pgen.1007745)
Supplement: S1 Table — (PDF) [file pgen.1007745.s014.pdf]

Table S1.

Sample	Name	Qpgraph clusters	Coverage x	Catalogue name	dd._mm._yyyy	Location	Location	Note on location	Provider	Total number of usable reads	Estimated error rates (%)	Heterozygosity estimates	Inbreeding coefficients	Number of segments	Mean length ROH	Median length ROH	Max length ROH	Total length ROH
Golden jackal	Golden Jackal	Golden Jackal	4,40	RKW 1332 ;SAMN03366713				Tel Aviv, Israel	Freedman et al. 2014	107048490	NA	0,00118						
Red wolf	Red wolf 3	Red Wolf	5,57	#652-M PDZA (SAMN10246099)	01._06._2004	29,9399086	-94,653957	"Rough location for capture zone of founders"; (from Point Defiance Zoo & Aquarium)	U.S. Fish and Wildlife Service	149530167	0,0756	0,00168	0,108					
Red wolf	Red wolf 2	Red Wolf	5,24	BioSample: SAMN02921318; Sample name: rwb; SRA: SRS661494		29,9399086	-94,653957	"Rough location for capture zone of founders"	vonHoldt et al. 2016	182476507	0,5853 **(*)**	0,00500	0,000					
Red wolf	Red wolf 1	Red Wolf	25,41	BioSample: SAMN02921317; Sample name: rwa; SRA: SRS661493		29,9399086	-94,653957	"Rough location for capture zone of founders"	vonHoldt et al. 2016	730585462	0,0403	0,00178	0,191					
Coyote	Florida	Coyote	6,96	BioSample: SAMN05439957; Sample name: FloridaCoyote; SRA: SRS1589014		30,1738346	-82,238964	"Estimated from vonHoldt et al. 2016"	vonHoldt et al. 2016	192248702	0,0395	0,00179	0,024					
Coyote	Illinois	Coyote	6,17	BioSample: SAMN02921303; Sample name: mwc; SRA: SRS661479		41,656687	-88,094688	"Estimated from vonHoldt et al. 2016"	vonHoldt et al. 2016	192453098	0,4814 **(*)**	0,00500	0,000					
Coyote	Missouri	Coyote	4,79	MSB:Mamm:58790 (SAMN10180423)	??._??._1987	37,186564	-93,603057	10 MI. W. SPRINGFIELD ON HWY 44	University of New Mexico Museum of Southwestern Biology (Gopalakrishnan et al. 2018)	120687507	0,0433	0,00155	0,020					
Coyote	Quebec	Coyote	3,27	BioSample: SAMN05736724; Sample name: QuebecWolf; SRA: SRS1677740		47,5618756	-75,394498	"estimated from vonHoldt et al. 2016"	vonHoldt et al. 2016	103090908	0,1931 **(*)**	0,00334	0,015					
Coyote	Alaska	Coyote	7,84	UAM:Mamm:19791 (SAMN10180421)	04._03._1991	67,73333333	-152,4166667	Confluence of John River and Hunt Fork	University of Alaska Museum of the North (Gopalakrishnan et al. 2018)	202147010	0,0473	0,00154	0,171					
Coyote	Mexico	Coyote	11,33	MSB:Mamm:83943 (SAMN10180422)	13._10._1996	28,9954	-112,38596	Isla Tiburon	University of New Mexico Museum of Southwestern Biology (Gopalakrishnan et al. 2018)	286356442	0,0420	0,00160	0,199					
Coyote	California	Coyote	26,64	BioSample: SAMN02921301; Sample name: cac; SRA: SRS661477		37,4486362	-120,581619	"Estimated from vonHoldt et al. 2016"	vonHoldt et al. 2016	811742421	0,0780	0,00191	0,146					
Coyote	Alabama	Coyote	5,06	BioSample: SAMN02921302; Sample name: alc; SRA: SRS661478		32,8918405	-86,081453	"Estimated from vonHoldt et al. 2016"	vonHoldt et al. 2016	167870483	0,4491 **(*)**	0,00500	0,000					
Eastern timber wolf	Algonquin 3	Eastern Timber Wolf	5,20	250514 (SAMN10246098)	03._02._2010	45,34459	-78,94058	Algonquin National Park	Ontario Ministry of Natural Recources	144748744	0,0405	0,00163	0,192					
Eastern timber wolf	Algonquin 2	Eastern Timber Wolf	4,21	BioSample: SAMN05791669; Sample name: AlgonquinWolf13467; SRA: SRS1704985		45,8371591	-78,3813126	Algonquin National Park	vonHoldt et al. 2016	112047899	0,0610	0,00166	0,097					
Eastern timber wolf	Algonquin 1		6,18	BioSample: SAMN05791668; Sample name: AlgonquinWolf13470; SRA: SRS1704945		45,8371591	-78,3813126	Algonquin National Park	vonHoldt et al. 2016	122850385	0,0455	0,00231	0,000					
Great lakes wolf	IRNP	Great Lakes Wolf	9,04	BioSample: SAMN04209256; Sample name: RKW1387; SRA: SRS1136754		47,9958654	-88,9092899	Isle Royale National Park	vonHoldt et al. 2016, Marsden et al. 2016	383060298	0,0681	0,00110	0,400	297	1730698	1468456	5353278	514017249
Great lakes wolf	Minnesota		23,82	BioSample: SAMN02921310; Sample name: glw; SRA: SRS661486		46,745947	-94,505756	"Estimated from vonHoldt et al. 2016"	vonHoldt et al. 2016, Marsden et al. 2016	829355849	0,0391	0,00182	0,122					
Wolf	Eurasia 2	Eurasian Wolf	7,72	GW2 ;SAMN01974487		65,6873915	156,7958972	Chukotka, Russia	Wang et al. 2013	211117215	0,0836	0,00138	0,185					
Wolf	Eurasia 1	Eurasian Wolf	9,56	GW1 ;SAMN01974486		50,7793339	82,4104216	Altai, Russia	Wang et al. 2013	292402755	0,1087	0,00159	0,129					
Wolf	Eurasia 3	Eurasian Wolf	9,83	GW3 ;SAMN01974488		53,2799469	34,2068799	Bryansk, Russia	Wang et al. 2013	329090300	0,1460	0,00148	0,166					
Wolf	Mexico 1	Mexican Wolf	22,15	"BioSample: SAMN02921314+SAMN02921315; Sample name: mxa+mxb; SRA: SRS661490+SRS661491"		34,2923957	-106,7932892	"Estimated from vonHoldt et al. 2016"	Fan et al. 2015	618720272	0,0976	0,00062	0,714	434	1671394	1446006	6784185	725385081
Wolf	Mexico 2	Mexican Wolf	2,08	MSB:Mamm:259087 (SAMN10180511)	02._06._2007	34,2923957	-106,7932892	Sevilleta National Wildlife Refuge	University of New Mexico Museum of Southwestern Biology (Gopalakrishnan et al. 2018)	56091351	0,0396	0,00112	0,370					
Wolf	St. Lawrence Island		11,82	UAM:Mamm:30678 (SAMN10246094)	26._04._1994	63,77972222	-171,7411111	St. Lawrence Island	University of Alaska Museum of the North	303885641	0,0415	0,00104	0,373					
Wolf	Pacific Coast		13,56	UAM:Mamm:21440 (SAMN10246091)	20._03._1992	56,75	-133,5	Alexander Archipelago	University of Alaska Museum of the North	345355315	0,0413	0,00128	0,220	105	1495222	1394477	2969331	156998277
Wolf	Yellowstone 2	Yellowstone Wolf	24,25	BioSample: SAMN02921320; Sample name: ysa; SRA: SRS661496		44,427963	-110,588455	Yellowstone National Park	Fan et al. 2015	680714876	0,0444	0,00500	0,166	106	1499363	1365943	3470279	158932467
Wolf	Yellowstone 1	Yellowstone Wolf	3,66	BioSample: SAMN02921322; Sample name: ysc; SRA: SRS661498		44,427963	-110,588455	Yellowstone National Park	Fan et al. 2015	189064388	0,6364 **(*)**	0,00149	0,049					
Wolf	Toronto	Atlantic, Alaskan, Central and Polar Wolf	8,24	2-PAM-720 (SAMN10246095)	??._??._2003	43,72	-79,55	Rexdale **(#)**	North American Fur Auctions	222358489	0,0549	0,00151	0,058					
Wolf	Atlantic Coast	Atlantic, Alaskan, Central and Polar Wolf	8,19	BAF-117 (SAMN10246086)	??._??._2002	54,9	-59,78	Postville	North American Fur Auctions	222609059	0,0611	0,00148	0,083					
Wolf	Alaska 1	Atlantic, Alaskan, Central and Polar Wolf	6,26	ARF07 (SAMN10246085)	01._09._2002	67	-158	Kobuk River	North American Fur Auctions	161650685	0,0495	0,00140	0,039					
Wolf	Alaska 2	Atlantic, Alaskan, Central and Polar Wolf	6,24	BioSample: SAMN05439956; Sample name: AlaskanWolf; SRA: SRS1589013		63,500043	-146,275791	"Estimated from vonHoldt et al. 2016"	vonHoldt et al. 2016	170693586	0,0406	0,00132	0,015					
Wolf	Saskatchewan	Atlantic, Alaskan, Central and Polar Wolf	4,63	GWC-857 (SAMN10246093)	??._??._2002	55,1	-105,28	La Ronge	North American Fur Auctions	134908797	0,0640	0,00156	0,005					
Wolf	Qamanirjuaq	Atlantic, Alaskan, Central and Polar Wolf	3,82	RI79 (SAMN10246092)	01._04._2000	64,43	-93,1	Tehery Lake	Department of Environment Nunavut	100312929	0,0463	0,00115	0,027					
Wolf	South Baffin	Polar, West and East Arctic Wolf	12,72	PG65 (SAMN10246088)	05._02._2000	65,17	-65,5	Mouth of Chidliak Fiord/Cumberland Sound	Department of Environment Nunavut	335568758	0,0434	0,00114	0,267					
Wolf	North Baffin	Polar, West and East Arctic Wolf	14,13	PI58 (SAMN10246087)	14._12._1999	72,7	-77,98	"Graveyard"	Department of Environment Nunavut	363205435	0,0402	0,00142	0,090	60	1382523	1255827	2684601	82951367
Wolf	Victoria Island	Polar, West and East Arctic Wolf	11,77	HW70 (SAMN10246096)	25._01._2004	72,77	-111,02	Wynniatt Bay	Environment and Natural Resources Northwest Territories	307779135	0,0393	0,00146	0,044					
Wolf	Banks Island	Polar, West and East Arctic Wolf	15,36	SW24 (SAMN10246089)	20._04._2004	74,13	-119,75	Thomsen River	Environment and Natural Resources Northwest Territories	400130823	0,0390	0,00137	0,140	91	1526788	1397649	3131701	138937717
Wolf	Krummelangsø		0,44	M10575 (SAMN10246097)	??._??._1995	74,08333	-23,83333	Krummelangsø	Natural History Museum of Denmark	159952327	0,1461 **(*)**	0,00500	0,041					
Wolf	Ellesmere 2	Polar, West and East Arctic Wolf	5,95	GF217 (SAMN10246090)	06._04._2002	76,42	-82,88	In community of Grise Fiord	Department of Environment Nunavut	153517018	0,0399	0,00125	0,036					
Wolf	Daneborg	Atlantic, Alaskan, Central and Polar Wolf / Polar, West and East Arctic Wolf	11,21	Daneborg (SAMN10180430)	13._01._2013	74,3	-20,2334697	Daneborg	Greenland Institute of Natural Resources (Gopalakrishnan et al. 2018)	284695953	NA	0,00102	0,313	202	1592261	1393555	4023046	321636675
Wolf	Ellesmere 1	Polar, West and East Arctic Wolf	7,69	GF212 (SAMN10180431)	24._03._2002	77,22	-85,42	10 km south of Gunnars Island	Department of Environment Nunavut (Gopalakrishnan et al. 2018)	203079413	0,0400	0,00084	0,385					

References

Freedman AH, et al. (2014) Genome sequencing highlights the dynamic early history of dogs. PLoS Genet 10(1):e1004016.
vonHoldt BM, et al. (2016) Whole-genome sequence analysis shows that two endemic species of North American wolf are admixtures of the coyote and gray wolf. Sci Adv 2(7):e1501714.
Wang G-D, et al. (2013) The genomics of selection in dogs and the parallel evolution between dogs and humans. Nat Commun 4:1860.
Fan Z, et al. (2016) Worldwide patterns of genomic variation and admixture in gray wolves. Genome Res 26(2):163–173.
Gopalakrishnan S, et al. (2018) Interspecific gene flow shaped the evolution of the genus Canis. Curr Biol (in press).
Marsden, CD, et al (2016) Bottlenecks and selective sweeps during domestication have increased deleterious genetic variation in dogs. Proc Natl Acad Sci U S A 113(1):152-157.

\# We acknowledge that the location of the sample seems highly improbable, however it is information we have been given along with the sample and therefore the information we present. While it is surprising, it is the only sample from this location or any large city location, which initially was why the sample was interesting. The specific sample originated from the North American Fur Auctions and there is a Hudson's Bay Auction House, Rexdale, Ontario, Toronto. The information given together with other samples from the North American Fur Auctions, fits a specific rural location with detail on nearby named locations. The gps location with the sample fits a green area along a river going through Rexdale. Perhaps the wolf truly came from here fowling the river. We are unable to conclude whether the sample originates from a rare incident or mislabelling from the provider, therefore we chose to present the metadata we were given with the sample.

* The specific samples are treated as high error rate samples.
